# Acid anion electrolyte effects on platinum for oxygen and hydrogen electrocatalysis

**DOI:** 10.1038/s42004-022-00635-1

**Published:** 2022-02-18

**Authors:** Gaurav Ashish Kamat, José A. Zamora Zeledón, G. T. Kasun Kalhara Gunasooriya, Samuel M. Dull, Joseph T. Perryman, Jens K. Nørskov, Michaela Burke Stevens, Thomas F. Jaramillo

**Affiliations:** 1 Department of Chemical Engineering, Stanford University, 443 Via Ortega, Stanford, CA 94305 USA; 2 SUNCAT Center for Interface Science and Catalysis, SLAC National Accelerator Laboratory, 2575 Sand Hill Road, Menlo Park, CA 94025 USA; 3 Catalysis Theory Center, Department of Physics, Technical University of Denmark, 2800 Kongens Lyngby, Denmark

**Keywords:** Electrocatalysis, Chemical engineering, Electrocatalysis, Energy

## Abstract

Platinum is an important material with applications in oxygen and hydrogen electrocatalysis. To better understand how its activity can be modulated through electrolyte effects in the double layer microenvironment, herein we investigate the effects of different acid anions on platinum for the oxygen reduction/evolution reaction (ORR/OER) and hydrogen evolution/oxidation reaction (HER/HOR) in pH 1 electrolytes. Experimentally, we see the ORR activity trend of HClO_4_ > HNO_3_ > H_2_SO_4_, and the OER activity trend of HClO_4_ $>$ HNO_3_ ∼ H_2_SO_4_. HER/HOR performance is similar across all three electrolytes. Notably, we demonstrate that ORR performance can be improved 4-fold in nitric acid compared to in sulfuric acid. Assessing the potential-dependent role of relative anion competitive adsorption with density functional theory, we calculate unfavorable adsorption on Pt(111) for all the anions at HER/HOR conditions while under ORR/OER conditions ${{{{{\rm{Cl}}}}}}{{{{{{\rm{O}}}}}}}_{4}^{-}$ binds the weakest followed by ${{{{{\rm{N}}}}}}{{{{{{\rm{O}}}}}}}_{3}^{-}$ and ${{{{{\rm{S}}}}}}{{{{{{\rm{O}}}}}}}_{4}^{2-}$. Our combined experimental-theoretical work highlights the importance of understanding the role of anions across a large potential range and reveals nitrate-like electrolyte microenvironments as interesting possible sulfonate alternatives to mitigate the catalyst poisoning effects of polymer membranes/ionomers in electrochemical systems. These findings help inform rational design approaches to further enhance catalyst activity via microenvironment engineering.

## Abstract (web summary)

Microenvironment engineering through electrolyte optimization is a promising approach to mitigate catalyst poisoning effects in electrochemical systems, but the role of electrolyte anions is not fully understood. Here, in a combined experimental-theoretical evaluation, the authors study the effects of different acidic electrolytes (pH 1) on platinum for hydrogen (HER/HOR) and oxygen electrocatalysis (ORR/OER), finding that oxygen reduction performance can be improved 4-fold using nitric rather than sulfuric acid.

## Introduction

Oxygen and hydrogen electrocatalysis play a key role in many promising renewable electrochemical energy storage and conversion technologies such as fuel cells and electrolyzers. Proton exchange membrane (PEM) fuel cells (FCs) and electrolyzers are particularly important in the transition to a sustainable hydrogen-based economy^1^ and significant effort has gone into understanding the performance of electrocatalysts used in such devices under diverse conditions^2^. There is an opportunity to continue to advance these and related technologies through a deeper fundamental understanding of electrolyte effects at the catalyst interface. This work probes such phenomena.

Platinum (Pt) is one of the most widely used and well-characterized catalyst materials for hydrogen and oxygen electrocatalysis in both fundamental and applied studies owing to its high activity and stability under acidic conditions^2–4^. Devices such as PEM hydrogen FCs, in particular, require significant amounts of Pt to perform the cathodic oxygen reduction reaction (ORR, O_2_ + 4*e*^–^ + 4H^+^ → 2H_2_O, E^0^ = 1.23 V_RHE_; 0.2–0.28 mg_Pt_ ${{{{{\rm{c}}}}}}{{{{{{\rm{m}}}}}}}_{{{{{{\rm{geo}}}}}}}^{-2}$ in a recent^5^ state-of-the-art device) and anodic hydrogen oxidation reaction (HOR, H_2_ → 2H^+^ + 2*e*^–^, E^0^ = 0 V_RHE_; 0.05–0.07 mg_Pt_ ${{{{{\rm{c}}}}}}{{{{{{\rm{m}}}}}}}_{{{{{{\rm{geo}}}}}}}^{-2}$ in a recent^5^ state-of-the-art device). PEM water electrolyzers, which also operate in acidic conditions to split water into O_2_ and H_2_ gas via the oxygen evolution reaction (OER, 2H_2_O → O_2_ + 4*e*^–^ + 4H^+^, E^0^ = 1.23 V_RHE_) and the hydrogen evolution reaction (HER, 2H^+^ + 2*e*^–^ → H_2_, E^0^ = 0 V_RHE_) at the anode and cathode, respectively, also use Pt as the primary catalyst component at the cathode (0.5–1 mg_Pt_ ${{{{{\rm{c}}}}}}{{{{{{\rm{m}}}}}}}_{{{{{{\rm{geo}}}}}}}^{-2}$ in recent^6^ state-of-the-art device). Finally, while Pt has not been commercialized for the OER, nor is it considered a highly active catalyst for the OER in acid, it is one of the few materials with significant stability to dissolution at such high oxidizing potentials^3,7–10^. Thus, adding studies of the OER on Pt to complement the more technologically relevant studies of HER, HOR, and ORR can provide a broader view of electrolyte effects on this important catalyst material for key energy conversion reactions. Fundamental understanding of the relationships between catalyst material, electrolyte composition, and catalytic performance is important to engineer ways to use electrolyte effects as a lever for tuning electrochemical system performance.

Microenvironment electrolyte/anion effects on electrocatalyst activity have been studied at a fundamental level for the ORR in several electrolytes on Pt and a few other catalysts^11–22^, while few studies have examined electrolyte effects for the OER^23^, HER^24–27^, and HOR^24–27^ on Pt^28–36^. Based on the better-known electrolyte-activity relationships for the ORR on Pt, perchloric acid (HClO_4_) has been noted as the standard^37^ electrolyte for benchmarking performance^16,38^. Previous work suggests that the high performance and stability of Pt in HClO_4_ is due to weak interactions between ${{\mbox{Cl}}}$ and the Pt surface^16,28,37,38^. In contrast, the presence of ion species in the double layer microenvironment has also been shown to negatively impact Pt activity for the ORR. Specifically, hydrogen halide acids (hydrochloric (HCl), hydrobromic (HBr), and hydroiodic (HI) acid)^13,39^ and other inorganic acids (sulfuric (H_2_SO_4_)^21,36,40,41^ and phosphoric (H_3_PO_4_)^16,17,41^ acid) have been shown to suppress Pt activity mainly due to competitive adsorption effects^28^. For the OER, previous experiments on Pt in HClO_4_ (~pH 0.1) and H_2_SO_4_ (~pH 0.3) suggest differences in activity and Tafel slope between the two electrolyte compositions and pH’s^23^. For the HOR, the suggested electrolyte-activity trend previously reported in acids of pH 0.3 on a rotating disk electrode (RDE) at 3600 rpm is as follows: HClO_4_ > H_2_SO_4_ > HCl^25^. Conversely for the HER, no significant activity shift has been observed regardless of electrolyte anion^24–27^. This negligible response to the electrolyte composition has been attributed to the large quantity of underpotentially deposited (UPD) hydrogen (H* adsorbed) that blocks anion adsorption prior to the HER^24–27^. Understanding electrolyte effects more broadly could help inform the design of higher performance systems. For example, although H_2_SO_4_ is known to actively poison the Pt surface during ORR^21,36,40,41^ and has moderate impact on OER and HOR catalysis^23–27^, Nafion^TM^, which contains similar sulfonate terminated sites anchored to a tetrafluoroethylene (PTFE) backbone that are also known to poison Pt^42–48^, is the most common polymer membrane and ionomer/binder used in PEM fuel cells and electrolyzers owing to its state-of-the-art proton conductivity. Another issue with modern PEMs is the generation of degradation products that, for instance, inhibit ORR activity and modify 4*e*^*–*^ selectivity through active site blocking and other related effects^49–51^. Previous studies have investigated the effects of various membrane degradation compounds and have elucidated how the specific geometric and chemical properties of these degradation products influences electrocatalytic processes^50–52^. Therefore, insight into how other anions in the surface microenvironment impact Pt activity could also advance rational design efforts for new polymer materials and interfaces that create enhanced microenvironments.

There are several near-surface phenomena^28,53,54^ that could be expected in the electrolyte double layer region that can affect catalyst activity^22,55,56^. Electrolyte effects have been proposed to impact the catalyst surface in a number of different ways, including but not limited to the following six interrelated phenomena that occur within the double layer microenvironment: (1) competitive adsorption, (2) potential drop redistribution, (3) adsorbate dipole moment/polarizability interaction, (4) ion-intermediate chemical interaction, (5) interfacial pH buffering, and (6) interfacial water structure alteration^28,53,54^. Competitive adsorption, one of the most commonly identified interactions, is not unique to electrocatalysis and has been well-characterized in thermal heterogeneous catalysis studies^57,58^. In electrochemistry, this involves non-reaction species, such as anions in the electrolyte phase, adsorbing to the surface and blocking active sites, possibly also altering the local electronic structure of neighboring active sites^28,53,54,59^. The interaction of anions with the surface occurs through chemisorption or physisorption depending on the magnitude of the adsorption free energy^28,53,54^, a phenomenon that we have previously^22^ explored with DFT calculations for other metals on the ORR. Potential drop redistribution^60^ refers to the potential drop across the electric double layer region changing due to the presence of adsorbed species under an applied external potential. This effect can be caused by adsorbate dipole moment/polarizability interactions^61^ that could alter the free energy of adsorbed intermediates as an external potential is applied^61^. Both anion-surface/anion-intermediate interactions and potential drop redistribution can be affected by the presence of anion species of varying identity as charged species play a role in screening the charge of the electrode as seen in models of the double layer region^62^. Electrolyte effects of this nature could contribute to changing the driving force for electron transfer at the electrode^53^, and therefore fundamental studies are needed to understand how to utilize this effect to induce a favorable enhancement of catalyst activity. Ion-intermediate chemical interactions refer to the stabilization of bound intermediates through the formation of complexes at the surface and have been mainly proposed for systems involving cations in alkaline electrolyte^63,64^. Other proposed effects include the ability of solvated ions to influence pH buffering near the electrode and the structure of the hydration shells around solvated ions changing the behavior and magnitude of interactions between solvated ions and reaction adsorbates and/or the catalyst surface^53,60^. However, solvation effects and interfacial water structure are difficult to measure experimentally and most reports of these phenomena originate from physics-based modeling of the near-electrode region^65^. In short, ions within the electrocatalyst microenvironment play an important role in modulating activity.

One common acid that remains underexplored for hydrogen and oxygen electrocatalysis on Pt is nitric acid (HNO_3_). Our previous work on Pd^22^, which is similar to Pt in that it binds oxygen adsorbates slightly more strongly than ideal^66^, indicates that HNO_3_ enhances ORR activity over H_3_PO_4_, as characterized by an onset potential improvement of 35 mV at –0.1 mA ${{{{{{\rm{cm}}}}}}}_{{{{{{{\rm{P}}}}}}}_{{{{{{\rm{d}}}}}}}}^{-2}$. In this work, we probe how anion identity (${{{{{\rm{N}}}}}}{{{{{{\rm{O}}}}}}}_{3}^{-}$, ${{{{{\rm{HS}}}}}}{{{{{{\rm{O}}}}}}}_{4}^{-}/{{{{{\rm{S}}}}}}{{{{{{\rm{O}}}}}}}_{4}^{2-}$, and ${{{{{\rm{Cl}}}}}}{{{{{{\rm{O}}}}}}}_{4}^{-}$) affects the performance of oxygen and hydrogen electrocatalysis on a well-defined Pt surface. We employ cyclic voltammetry (CV) to study HOR, HER, OER, and ORR activity on a polycrystalline Pt disk in pH 1 HClO_4_, H_2_SO_4_, and HNO_3_ electrolytes. We find clear differences in ORR and OER activity trends across the three electrolytes but similar performance in HER and HOR which could be attributed to the distinct potential ranges of the four reactions impacting the strength of the anion-catalyst and anion-intermediate interactions. Notably, we demonstrate that ORR performance can be improved 4-fold (at 0.9 V_RHE_) in nitric acid compared to in sulfuric acid. We deconvolute the role of anion competitive adsorption via density functional theory (DFT) modeling. Combining theory and experiment, we gain insight into physical phenomena in the microenvironment leading to the observed reaction activity and hypothesize that differences in the strength of anion-catalyst or anion-intermediate interactions are partially responsible for variance in activity in the higher OER/ORR potential ranges whereas the strength of such interactions is weaker near the lower HER/HOR potential range. Our fundamental approach to understanding microenvironment-activity relationships serves as a foundation to investigate more complex double-layer microenvironment interactions, ultimately guiding microenvironment engineering. From these findings, the possibilities remain open for the development of novel PEMs with weakly-adsorbing group terminations, such as nitrates and perchlorates, that could maintain high ionic conductivity, enable a more favorable reaction microenvironment, and limit degradation.

## Results

### Ex situ and in situ characterization of the Pt disk electrode

Investigating the relationship between catalyst and electrolyte composition can give important insights about how to engineer the double layer microenvironment of electrocatalysts to tune performance. We investigate the activity of the HOR, HER, OER, and ORR on a well-characterized (Supplementary Fig. 1) polished (roughness factor = 1.002 and root-mean-squared roughness = 4.56 nm by atomic force microscopy (AFM)) polycrystalline Pt disk (0.196 ${{{{{\rm{c}}}}}}{{{{{{\rm{m}}}}}}}_{{{{{{\rm{geo}}}}}}}^{2}$) in 0.1 M (pH 1) HClO_4_, H_2_SO_4_, and HNO_3_ electrolytes (made of the highest purity commercially available, see “Methods: Electrolyte preparation”) with an RDE setup (1600 rpm, 20 mV s^–1^ CV in N_2_/H_2_/O_2_; CV(s) used interchangeably for cyclic voltammetry/voltammogram(s)). Electrochemical testing for oxygen and hydrogen electrocatalysis is performed after rigorous electrode conditioning according to standardized protocols^37,67^ (see the “Methods section”, Supplementary Fig. 2a, and Supplementary Note 1). For continuous, dense, and planar surfaces, AFM is the most direct method to understand the real exposed catalyst surface area^22,68–71^. Electrochemical surface area (ECSA) estimation by hydrogen under-potential deposition (H_UPD_) methods is standard^37,67^, but electrolyte composition and surface structure can complicate such analysis (see the “Methods” section, Supplementary Fig. 2b, and Supplementary Note 1). Therefore, to the best of our ability, we have determined that the geometric electrode area is approximately equal to the real exposed catalyst (Pt) surface in the three electrolytes indicating that performance changes are likely not due to surface area disparities. Hence, we simply normalize by ${{{{{\rm{c}}}}}}{{{{{{\rm{m}}}}}}}_{{{{{{\rm{Pt}}}}}}}^{2}$ as measured by post-test AFM. Physical characterization of the Pt disk before and after electrochemical experiments is available/discussed in Supplementary Fig. 1 and Supplementary Note 1.

### Characteristic electrochemical evaluation of Pt in HClO_4_, H_2_SO_4_, and HNO_3_

**Fig. 1:**
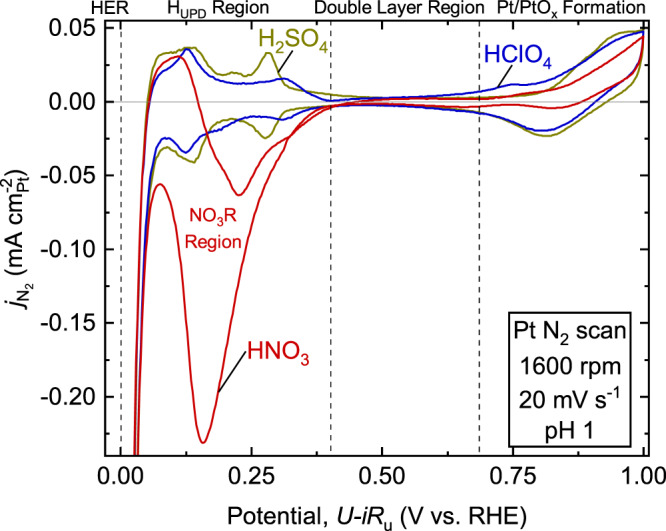
Characteristic Pt cyclic voltammogram in N_2_-saturated HClO_4_, H_2_SO_4_, and HNO_3_. Representative cyclic voltammogram (CV) of the Pt disk at 20 mV s^–1^ in N_2_-sat’ed HClO_4_, H_2_SO_4_, and HNO_3_. Dashed regions indicate approximate divisions of potential ranges as indicated along the top: hydrogen evolution reaction (HER) region, hydrogen under-potential deposition (H_UPD_) region, double layer capacitance region, and surface Pt oxidation/PtO_*x*_ reduction region. Color code: HClO_4_ (blue), HNO_3_ (red), and H_2_SO_4_ (olive).

The characteristic redox profiles, the CVs collected in N_2_ for the Pt disk in each electrolyte shown in Fig. 1, elucidate certain surface processes or competing reactions in each electrolyte environment. The region between ~0–0.4 V_RHE_ corresponds to the H_UPD_ processes^72^, and platinum in HClO_4_ acid typically exhibits two oxidation and reduction peaks in this region, corresponding to adsorption and desorption of H^8^. Similar features are also seen in H_2_SO_4_, although these are more pronounced and negatively shifted relative to those seen in HClO_4_ owing to the ${{{{{\rm{HS}}}}}}{{{{{{\rm{O}}}}}}}_{4}^{-}/{{{{{\rm{S}}}}}}{{{{{{\rm{O}}}}}}}_{4}^{2-}$ anions affecting the adsorption/desorption of hydrogen^40,41,73,74^. Notably, Pt in nitric acid displays a single large reduction feature, not seen in the other two electrolytes, previously attributed to nitrate reduction (NO_3_R) competing with H_UPD_ in this region and being ultimately suppressed by H_UPD_ and the HER as the potential decreases^75,76^. The suppression of NO_3_R by H_UPD_/HER under an inert environment supports the hypothesis that it would likely be suppressed in H_2_-saturated electrolyte during the HOR/HER. As NO_3_R does occur within the ORR potential window, specifically in the lower end of the mass transport limited region (away from practical applied potentials in devices), it is possible that NO_3_R is also partly suppressed during ORR catalysis. However, to not overestimate our ORR current, in our analysis below we have corrected for its possible contribution with a N_2_-CV subtraction. After cycling in N_2_/H_2_/O_2_ and after a separate 10 min constant potential hold at 0.16 V_RHE_, we do not observe any ammonia, a possible NO_3_R product^75,77^, in solution, suggesting it is present below detection limits (<5${{\upmu }}$M) or is quickly oxidized back to nitrate (see nuclear magnetic resonance (NMR) spectroscopy analysis^78^ of the electrolyte after testing in Supplementary Fig. 3). The region between 0.4 V_RHE_ and 0.6 V_RHE_ is the double layer capacitive charging region where no Faradaic processes occur^37^. The region above 0.6 V_RHE_ shows a small redox feature which has been attributed to surface Pt oxidation and reduction, which is thought to be essential in conditioning the surface for electrocatalysis (see N_2_-CVs with a higher potential range in Supplementary Fig. 2)^37^. The analysis of these features across different potential ranges is generally also relevant to understanding the effects of other dissolved species in the electrolyte such as those originating from polymer degradation in the presence of an ionomer^49^.

### Anion effects on Pt during oxygen and hydrogen electrocatalysis in HClO_4_, H_2_SO_4_, and HNO_3_

**Fig. 2:**
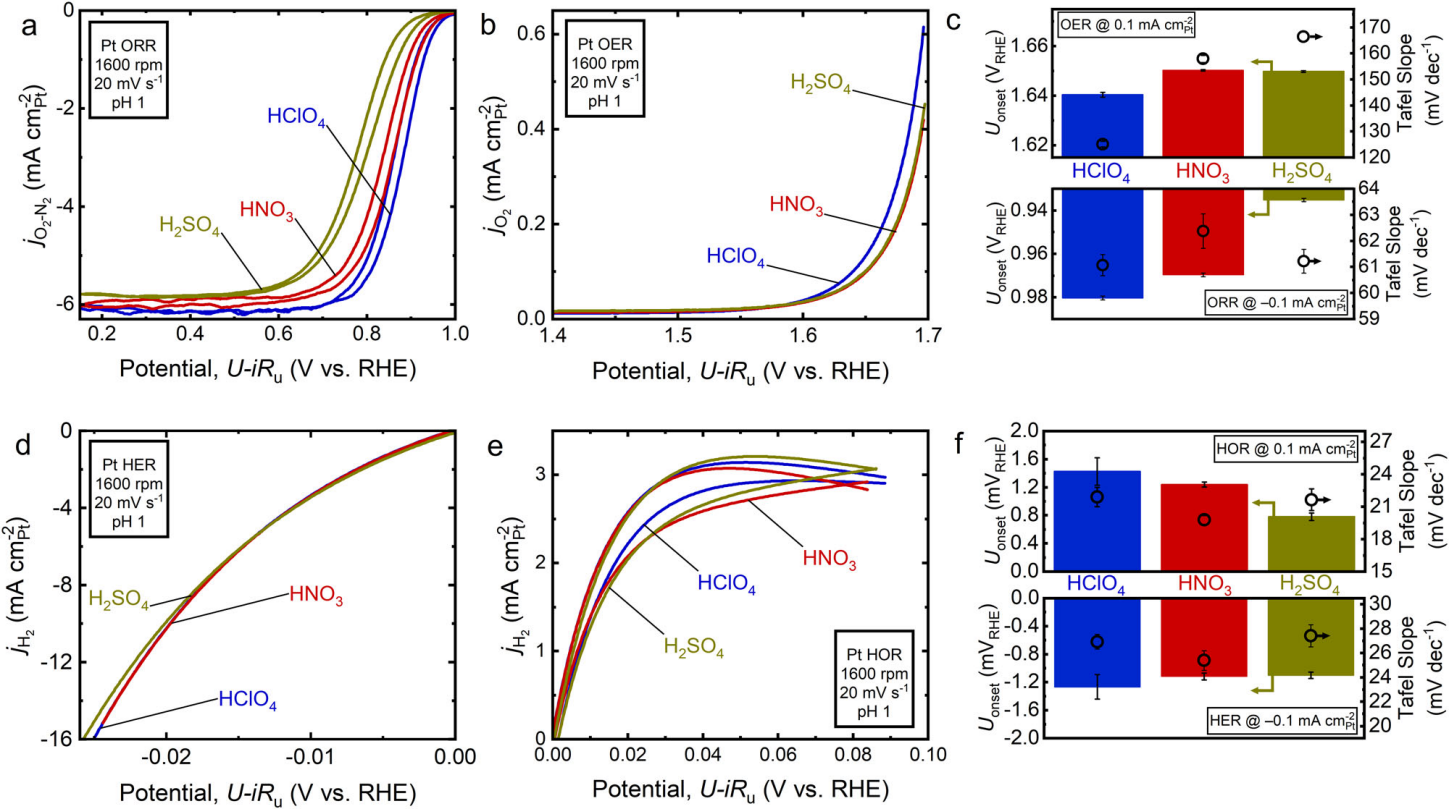
Evaluation of Pt for oxygen and hydrogen electrocatalysis in HClO_4_, HNO_3_, and H_2_SO_4_. **a** Representative third-cycle-averaged N_2_-subtracted oxygen reduction reaction (ORR) rotating disk electrode (RDE) cyclic voltammogram (CV) of the Pt disk in pH 1 HClO_4_, HNO_3_, and H_2_SO_4_. **b** Representative second-cycle-averaged oxygen evolution reaction (OER) RDE CV of the Pt disk in pH 1 HClO_4_, HNO_3_, and H_2_SO_4_ (OER trend is consistent as a function of cycle, Supplementary Fig. 4). **c** Average OER (top) and ORR (bottom) onset potential (bars, left *y*-axis) $\pm$0.1 mA ${{{{{{\rm{cm}}}}}}}_{{{{{{\rm{geo}}}}}}}^{-2}$ for all three acids and average Tafel slope (circles, right *y*-axis) in the low current density region. **d** Representative third-cycle-averaged hydrogen evolution rection (HER) RDE CV of the Pt disk in pH 1 HClO_4_, HNO_3_, and H_2_SO_4_. **e** Representative third-cycle-averaged hydrogen oxidation reaction (HOR) RDE CV of the Pt disk in pH 1 HClO_4_, HNO_3_, and H_2_SO_4_. **f** Average HOR (top) and HER (bottom) onset potential (bars, left *y*-axis)$\pm$0.1 mA ${{{{{{\rm{cm}}}}}}}_{{{{{{\rm{geo}}}}}}}^{-2}$ for all three acids and average Tafel slope (circles, right y-axis) in the low current density region. Error bars represent the standard deviation of separate triplicate measurements. All CVs collected at 1600 rpm, 20 mV s^–1^, and *iR*_u_ corrected. Color code: HClO_4_ (blue), HNO_3_ (red), and H_2_SO_4_ (olive).

We summarize our hydrogen and oxygen electrocatalysis evaluation of Pt in 0.1 M HClO_4_, H_2_SO_4_, and HNO_3_ in Fig. 2a–f (see tabulated average performance metrics in Supplementary Table 1). Examining oxygen electrocatalysis first, it is clear the electrolyte plays an important role in modulating activity. Figure 2a–c displays the ORR and OER (also see Supplementary Fig. 4) measurements on the Pt disk in each of the three acid electrolytes. Figure 2a shows the average ORR polarization curves in the three acids, where HClO_4_ has the best performance, followed by HNO_3_ and then H_2_SO_4_, with onset potentials (at –0.1 mA ${{{{{\rm{c}}}}}}{{{{{{\rm{m}}}}}}}_{{{{{{\rm{Pt}}}}}}}^{-2}$) of 0.980 V_RHE_, 0.969 V_RHE_, and 0.935 V_RHE_, respectively. Pt in HClO_4_ for the ORR is a particularly well-studied system and the kinetic current density at 0.9 V_RHE_ is often used as a benchmark to ensure the catalyst and experimental setup are clean and reproducible^37^. On average, in our HClO_4_ measurements, the anodic sweep of the CV had a kinetic current density at 0.9 V_RHE_, as calculated by the Koutecký-Levich equation^79^ based on an AFM-based surface area normalization, of around –3.34 mA ${{{{{{\rm{cm}}}}}}}_{{{{{{\rm{Pt}}}}}}}^{-2}$ and the cathodic sweep around –1.68 mA ${{{{{{\rm{cm}}}}}}}_{{{{{{\rm{Pt}}}}}}}^{-2}$. When the values of both sweeps are averaged, the kinetic current density at 0.9 V_RHE_ is –2.52 mA ${{{{{{\rm{cm}}}}}}}_{{{{{{\rm{Pt}}}}}}}^{-2}$, which is well within the expected range, and is of greater magnitude than the 2 mA ${{{{{{\rm{cm}}}}}}}_{{{{{{\rm{Pt}}}}}}}^{-2}$ requirement^37^ for this measurement. Because impurities have been known to decrease the performance of the ORR, this suggests that our experimental setup had an adequate cleaning and Pt conditioning protocol applied and that measurements taken in other the electrolytes are reliable^37,67^. Looking at the other two acids, the average kinetic current density at 0.9 V_RHE_ is –1.36 mA ${{{{{{\rm{cm}}}}}}}_{{{{{{\rm{Pt}}}}}}}^{-2}$ for HNO_3_ and –0.35 mA ${{{{{{\rm{cm}}}}}}}_{{{{{{\rm{Pt}}}}}}}^{-2}$ for H_2_SO_4_. While Pt ORR activity in nitric acid has not been reported to our knowledge, a literature value for kinetic current density at 0.9 V_RHE_ in H_2_SO_4_^21^ is around –0.2 mA ${{{{{{\rm{cm}}}}}}}_{{{{{{\rm{Pt}}}}}}}^{-2}$, which is in close agreement with our measurement. Interestingly, the magnitude of the mass transfer limited current density is highest in HClO_4_ followed by in HNO_3_ and H_2_SO_4_, though this could be attributed to minor differences in oxygen solubility and electrolyte viscosity, which are both accounted for in the kinetic current density calculations^79–81^. Another observation is that the hysteresis between the anodic and cathodic sweeps of the CV is similar for HClO_4_ and HNO_3_ but has slightly more tapered shape for H_2_SO_4_, which exhibits more pronounced hysteresis than the other two in the kinetically-controlled region. This suggests the dynamics of ${{{{{\rm{Cl}}}}}}{{{{{{\rm{O}}}}}}}_{4}^{-}$ and ${{{{{\rm{N}}}}}}{{{{{{\rm{O}}}}}}}_{3}^{-}$ interacting with the Pt surface during ORR as a function of potential are similar^41^. In contrast, the increased hysteresis seen in H_2_SO_4_ could suggest that relative to ${{{{{\rm{Cl}}}}}}{{{{{{\rm{O}}}}}}}_{4}^{-}$ and ${{{{{\rm{N}}}}}}{{{{{{\rm{O}}}}}}}_{3}^{-}$, the ${{{{{\rm{HS}}}}}}{{{{{{\rm{O}}}}}}}_{4}^{-}/{{{{{\rm{S}}}}}}{{{{{{\rm{O}}}}}}}_{4}^{2-}$ microenvironment is more conducive to larger surface oxide coverages in the cathodic scan and/or, as previously reported^21,36,40,41^, that these anions interact more strongly with the Pt surface^37^. This is consistent with H_2_SO_4_ producing the lowest activity of the three acids and highlights HNO_3_ as a viable HClO_4_ alternative to study ORR as trace Cl^–^ in HClO_4_ results^37^ in significant decreases in measured activity, which can obscure experimental results.

**Fig. 3:**
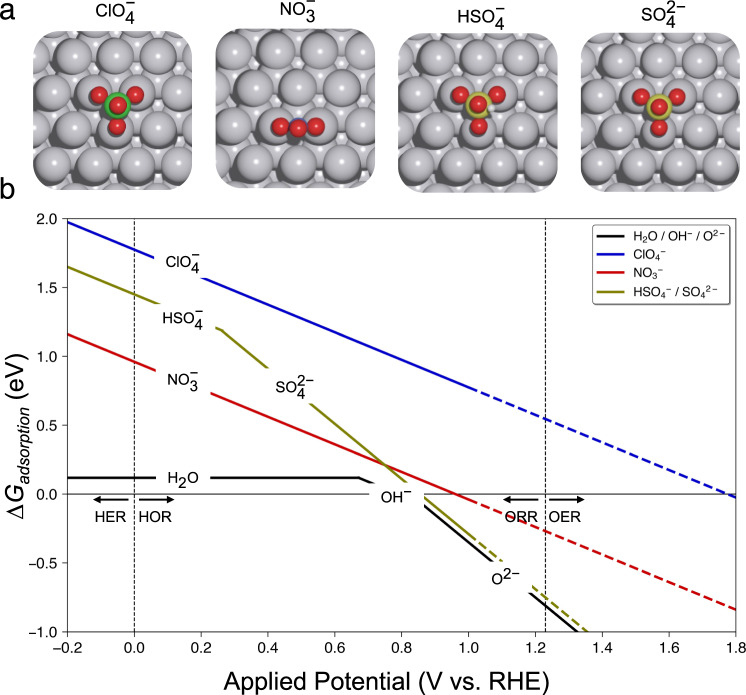
Potential dependent anion adsorption on Pt(111) surface. **a** The most stable adsorption configurations of anions on the Pt(111) surface. Color coded: O: red, H: white, Cl: green, N: blue, and S: olive. **b** The adsorption free energies of the anions, Δ*G*_adsorption_(A^*n*−^) (eV), on the Pt(111) surface as a function of applied potential (V vs. RHE). The colored dashed lines qualitatively shows the trends in Δ*G*_adsorption_(A^*n*−^) at higher potentials relevant for the oxygen evolution reaction (OER). Since the Pt(111) surface undergoes surface oxidation at these high potentials, we further calculated Δ*G*_adsorption_(A^*n*−^) trends on a PtO_2_(110) surface as shown in Supplementary Fig. 5 and Supplementary Table 4. Anion species color-coded: black (H_2_O/OH^–^/O^2–^), blue (${{{{{{\rm{ClO}}}}}}}_{4}^{-}$), red (${{{{{\rm{N}}}}}}{{{{{{\rm{O}}}}}}}_{3}^{-}$), and olive (${{{{{{\rm{HSO}}}}}}}_{4}^{-}/{{{{{\rm{S}}}}}}{{{{{{\rm{O}}}}}}}_{4}^{2-}$). HER/HOR, and ORR stand for hydrogen evolution/oxidation reaction and oxygen reduction reaction, respectively.

The increase in ORR overpotential in HNO_3_ and H_2_SO_4_ compared to HClO_4_ could be indicative of surface phenomena, within the ORR kinetic potential range, that interfere with adsorption of intermediates and leads to lower activity. Such phenomena would support the hypothesis, previously presented in literature^16,37,38^, that ${{{{{\rm{Cl}}}}}}{{{{{{\rm{O}}}}}}}_{4}^{-}$ does not interact strongly with catalyst materials during the ORR resulting in HClO_4_ demonstrating the highest ORR activity compared to other investigated electrolytes. To assess the strength of anion-surface interactions, we calculated the adsorption free energy of ${{{{{\rm{Cl}}}}}}{{{{{{\rm{O}}}}}}}_{4}^{-}$, ${{{{{\rm{N}}}}}}{{{{{{\rm{O}}}}}}}_{3}^{-}$, ${{{{{\rm{HS}}}}}}{{{{{{\rm{O}}}}}}}_{4}^{-}/{{{{{\rm{S}}}}}}{{{{{{\rm{O}}}}}}}_{4}^{2-}$, and H_2_O/OH^–^/O^2–^ on a Pt(111) surface as a function of applied potential with DFT (see more theory details in Supplementary Figs. 5, 6 and Tables 2–5). Figure 3 demonstrates that ${{{{{\rm{Cl}}}}}}{{{{{{\rm{O}}}}}}}_{4}^{-}$ has the weakest adsorption energy in the ORR kinetic and mixed kinetic-diffusion region (above 0.8 V_RHE_) followed by ${{{{{\rm{N}}}}}}{{{{{{\rm{O}}}}}}}_{3}^{-}$ and ${{{{{\rm{S}}}}}}{{{{{{\rm{O}}}}}}}_{4}^{2-}$. The calculated strong adsorption of ${{{{{\rm{S}}}}}}{{{{{{\rm{O}}}}}}}_{4}^{2-}$ anions on Pt(111) surface (Fig. 3) supports the hypothesis that competitive adsorption is likely lowering ORR activity in H_2_SO_4_ electrolyte compared to in HNO_3_ and HClO_4_. We employ a Tafel slope analysis of the ORR low current density region (Fig. 2c) to assess whether a change in reaction mechanism could explain this activity shift between the investigated electrolytes. The Tafel slopes for all three electrolytes are close to the 60 mV dec^–1^ value that is postulated in simulations and observed experimentally^82–84^, likely indicating that a mechanism change is not responsible for the ORR activity differences of Pt in HClO_4_, HNO_3_, and H_2_SO_4_. Subsequently, the observed differences in activity likely arise from the differences in the ${{{{{\rm{Cl}}}}}}{{{{{{\rm{O}}}}}}}_{4}^{-}$, ${{{{{\rm{N}}}}}}{{{{{{\rm{O}}}}}}}_{3}^{-}$, ${{{{{\rm{HS}}}}}}{{{{{{\rm{O}}}}}}}_{4}^{-}/{{{{{\rm{S}}}}}}{{{{{{\rm{O}}}}}}}_{4}^{2-}$ anion physical properties and their intrinsic behavior in the double layer microenvironment. In addition to possible competitive anion adsorption, which has been shown for ${{{{{\rm{HS}}}}}}{{{{{{\rm{O}}}}}}}_{4}^{-}/{{{{{\rm{S}}}}}}{{{{{{\rm{O}}}}}}}_{4}^{2-}$^21,36,40^, differences in the local electrostatic interactions, arising from the dipole moments of the anions, with the ORR intermediates may modulate the adsorption free energy of ORR intermediates^28,53,54,61,85,86^. Examining the chemical bonds within the tested anions, the electronegativities of Cl and O atoms are relatively similar so the dipole moment along the Cl–O bond is weak in magnitude. In contrast, the N–O bond has a larger dipole moment magnitude and the S–O bond should have the largest dipole moment of the three. It may be the case that larger dipole moments along bonds, even though the anions themselves have no net dipole owing to their tetrahedral or trigonal planar symmetry, can interact with adsorbed intermediates and change their binding energy to lower the observed activity^53,54,59,87^. However, the interaction of these dipole moments with the solvating water molecules and the formal charge on the anion (e.g. ${{{{{\rm{S}}}}}}{{{{{{\rm{O}}}}}}}_{4}^{2-}$ vs. ${{{{{\rm{HS}}}}}}{{{{{{\rm{O}}}}}}}_{4}^{-}$) could be confounding factors in this analysis^53,60,65^. Challenges in understanding these subtle, yet important effects serve as an excellent opportunity for future work to gain fundamental insight on how better tune the microenvironment interface.

Figure 2b shows the average (second cycle, cathodic/anodic averaged) CV performance of the Pt disk during OER in each of the three electrolytes. In the CVs, perchloric acid demonstrates the best performance with HNO_3_ and H_2_SO_4_ having similar behavior. Specifically, Fig. 2c shows the measured OER onset potential at +0.1 mA $\,{{{{{{\rm{cm}}}}}}}_{{{{{{\rm{Pt}}}}}}}^{-2}$ in HClO_4_, HNO_3_, and H_2_SO_4_ at 1.64, 1.65, and 1.65 V vs. RHE respectively, with the values in HClO_4_ and H_2_SO_4_ being within the expected range seen in the literature^7,23^. Notably, as seen in Supplementary Fig. 4, Pt OER activity decreases as a function of CV cycle in all of the electrolytes tested. This could be due to gradual oxidation of the initially reduced Pt surface at this high potential range^3,26,88^. In Fig. 2c, we see Pt OER Tafel slopes of ~125 mV dec^–1^ in HClO_4_ and ~160–170 mV dec^–1^ in HNO_3_ and H_2_SO_4_. This difference in the Tafel slope suggests changes in adsorbates, rate-limiting steps, and/or mechanism between better-performing HClO_4_ and HNO_3_ and H_2_SO_4_. While, no significant oxidation is seen in ex situ X-ray photoelectron spectra/spectroscopy (XPS) post-electrocatalysis (Supplementary Figure 1b), in the characteristic CV profiles (Fig. 1), there is an oxidation feature around 0.9–1.1 V vs. RHE in all electrolytes that has been attributed to OH/O adsorption^8,41,72^ to the Pt (see below for further discussion), suggesting that in situ the Pt surface for OER starts at least partially oxidized and likely continues oxidizing during catalysis.

Measuring the charge passed at the electrode just before the onset of the OER is one means to understand anion adsorption, a non-faradaic process that can have implications on OER catalysis, e.g. through coverage effects, adsorbate-adsorbate interactions, etc. While in situ spectroscopy and/or microscopy would be necessary to directly correlate the non-faradic current to anion adsorption, integrating the first cycle of each CV in the three acids from 1.4 V vs. RHE to the OER onset potential can yield valuable insights. Charge passed values of 74.1 ± 0.3 μC cm^–2^, 83.1 ± 0.5 μC cm^–2^, and 83.0  ± 0.3 μC cm^–2^ were calculated for perchloric, nitric, and sulfuric acids, respectively. Less charge passed in perchloric acid may indicate less anion adsorption versus oxide coverage, which could in turn allow for better OER activity owing to more available active sites^89,90^. Similarly, nitric and sulfuric acids demonstrate quantitatively similar amounts of charge passed and accordingly have nearly identical OER activity profiles and onset potentials. We also note that the Pt OER activity decreases with cycling; however, the activity trends remain the same (Supplementary Fig. 4). Due to the experimentally observed highly dynamic nature of the Pt surface, indicated by activity changes in OER with increased cycling and based on the computational Pourbaix diagram of Pt (Supplementary Fig. 7), DFT calculations on the Pt(111) surface may not be relevant at such high potentials (>1.0 V vs. RHE). Although the exact Pt surface structure/oxidation during OER cycling is not known experimentally, DFT calculations in the OER potential region were performed on a PtO_2_(110) surface to model anion adsorption trends on Pt-oxide (Supplementary Fig. 5 and Supplementary Table 4). We used a dashed line for the OER region in Fig. 3 to visually show that the trends in anion adsorption free energies in this region still hold qualitatively as shown by the calculations on PtO_2_(110) in Supplementary Table 4. While non-adsorbed anion-intermediate interactions such as electrostatic interactions (e.g. anion-induced dipole moments effects) are possible, competitive adsorption effects likely dominate in the OER potential region owing lower anion adsorption free energies with increasing applied potential (Fig. 3 and Supplementary Fig. 5 and Supplementary Table 4). The Pt OER activity trend of HClO_4_ $>$ HNO_3_ ~ H_2_SO_4_ can be explained by weaker adsorption of ${{{{{\rm{Cl}}}}}}{{{{{{\rm{O}}}}}}}_{4}^{-}$ compared to ${{{{{\rm{N}}}}}}{{{{{{\rm{O}}}}}}}_{3}^{-}$ and ${{{{{\rm{S}}}}}}{{{{{{\rm{O}}}}}}}_{4}^{2-}$ (Fig. 3 and Supplementary Fig. 5 and Supplementary Table 4).

To further probe the effects of electrolyte anions in important hydrogen fuel cell and water electrolyzer reactions, we systematically investigated the HER and HOR (Fig. 2d–f) in the three electrolytes. Figure 2d, e shows the CVs obtained for the HOR/HER, and although there are small statistical differences, the performance in all three electrolytes is nearly identical within the tested potential range. Although at higher HER current densities (<~10 mA ${{{{{\rm{c}}}}}}{{{{{{\rm{m}}}}}}}_{{{{{{\rm{Pt}}}}}}}^{-2}$) the overall CV performance in H_2_SO_4_ starts to decrease compared to that in HClO_4_ and HNO_3_, Fig. 2d, f indicates that at moderate current densities HER performance is very similar across the three electrolytes. Moreover, the onset potentials (Fig. 2f) in the three electrolytes for HER and HOR as measured at –0.1 mA ${{{{{{\rm{cm}}}}}}}_{{{{{{\rm{Pt}}}}}}}^{-2}$ and +0.1 mA ${{{{{{\rm{cm}}}}}}}_{{{{{{\rm{Pt}}}}}}}^{-2}$, respectively, vary by no more than 1 mV from each other as a function of electrolyte. This observation can be explained by the significantly high anion adsorption free energies (Fig. 3) corresponding to unfavorable adsorption on Pt(111) at the lower HER/HOR potential range compared to ORR and OER. We further verified this trend by performing calculations on a 1 monolayer (ML) H*-Pt(111) surface with van der Waals (vdW) corrections^91^ (Supplementary Fig. 6 and Tables 5–6). It is important to note that the charge state of the anions is an important factor especially for the hydrogen-covered surfaces due to the limited interaction with the surface and the resulting limited charge-redistribution (Supplementary Table 7). Moreover, the HER and HOR generally involve smaller adsorbates such as *H and *H_2_ that have weak dipoles^92^ and thus do not respond as easily to electric field effects and anion dipole moments^53,61^. We hypothesize that the chemical nature of the HER/HOR intermediates, along with weaker anion interactions with the catalyst surface, as supported by DFT calculations, both contribute to the observation of similar hydrogen electrocatalysis activity in all three electrolytes. Figure 2f also displays the Tafel slopes of the HER and HOR where, in addition to the highly similar onset potentials, the average Tafel slopes for both reactions are within ~2.1 mV dec^–1^ of each other suggesting no significant change in mechanism and overall performance between the electrolytes in the tested potential range. In short, we believe the differences in potential range and chemical nature of reaction intermediates result in the observed activity trend for ORR/OER and lack thereof for HER/HOR.

## Discussion

Electrolyte effects on electrochemical performance is an area of active research that could produce transformative breakthroughs for renewable energy devices such as fuel cells and electrolyzers in the future by potentially enhancing the activity of existing catalysts past present limitations. We demonstrate in this work that the intrinsic performance of Pt for oxygen and hydrogen electrocatalysis can be modulated via electrolyte choice and potential tuning. We report the intrinsic oxygen and hydrogen electrocatalysis performance of Pt in nitric acid which, to our knowledge, remains underreported in the literature. We also demonstrate that Pt ORR activity in nitric acid is 4$\times$ that of Pt in sulfuric acid at 0.9 V_RHE,_ which is the most chemically analogous electrolyte to the sulfonic acid environment of Nafion (or similar) PEM membranes and/or ionomers. Specifically, oxygen electrocatalysis behaves differently across electrolytes due to taking place in a potential range in which anions interact with Pt more strongly, and likely due to having more complex intermediate species. In contrast, hydrogen electrocatalysis demonstrates similar performance between electrolytes within the tested potential range. One such possibility of anion-intermediate interaction effects involves OER and ORR adsorbates such as *OOH and *H_2_O_2_ having a greater capacity to respond to external electric fields compared to reaction intermediates involved in HER/HOR. For the HER and HOR, a combination of weaker anion–catalyst and/or anion–intermediate interactions at more negative potentials and intermediates such as *H generating a smaller dipole moment^92^ likely prohibit the same magnitude of anion effects on activity. Rigorous *operando* studies, tracking adsorbed surface and double layer species, would help probe this hypothesis and provide further physical insight into electrolyte/anion effects. Analysis of the surface after electrocatalysis reveals negligible changes, indicating that activity trends arise from the intrinsic properties and double layer microenvironment behavior of the different anions. Future work in this area could be performed for more complex catalyst and/or electrolyte formulations and in full device conditions, for example with an ionomer and PEM present.

We have shown that the electrocatalytic performance of Pt depends on the local potential-dependent microenvironment in which anions play important roles. Moreover, it is well-known that electrochemical devices/experiments that employ PEM membranes and/or ionomer in Pt catalyst ink formulations can suffer from active site poisoning from the sulfonate chain terminal groups in commonly used PEM polymers (i.e. Nafion)^44,45,47,48^. This is consistent with the observed poor performance of Pt in sulfuric acid compared to nitric acid (this work) and perchloric acid (this work and others^21,36,40,41^). We propose that polymer engineering of PEM membranes and, especially of ionomers, which are in the most contact with the catalyst, may help mitigate poisoning and perhaps even enhance the local catalyst microenvironment. In addition, the synthesis of new membrane compositions could mitigate previously observed ORR activity losses owing to degradation products if these new membranes are more chemically stable in the presence of damaging species such as peroxides^49,51,52^. For example, our results motivate the design of PEM membranes and ionomers that are terminated with species more similar to nitrate and perchlorate as opposed to sulfate-like (e.g. sulfonate) species. Our work encourages the engineering of new polymer electrolytes and binders to enhance reaction rates via microenvironment engineering without needing changes in catalyst material.

## Methods

### Pt disk preparation

In this study, we used a metallic platinum disk (0.196 ${{{{{\rm{c}}}}}}{{{{{{\rm{m}}}}}}}_{{{{{{\rm{geo}}}}}}}^{2}$; TANAKA Precious Metals) manually polished with aluminum oxide slurry (Allied High Tech Products) of varying sizes as recommended in literature^37^. Beginning with a 5 μm slurry dispersed on a Nylon pad, we moved the disk surface on a polishing pad for 5 min in a figure-8 pattern. Following this, the same procedure was repeated with a 0.3 μm slurry on a microfiber cloth surface and then a 0.05 μm slurry on a microfiber cloth surface. AFM (see methods below) measurements indicate a roughness factor around 1.001 and root mean squared roughness of 4.19 nm. This Pt disk was stored in Millipore water (*R* = 18.2 MΩ cm) when not in use and was polished and flame annealed prior to each RDE experiment. In the initial stages of this work, we attempted using physical vapor deposited Pt thin films (with and without a Ti sticking layer) onto glassy carbon disk inserts but the films showed poor adhesion, delaminating within seconds or minutes after submersion in 0.1 M strong acid electrolyte.

### Electrolyte preparation

We prepared 0.1 M (pH 1) electrolyte solutions for three strong acids using new stock solutions of the highest purity commercially available: perchloric (99.999% trace metals basis, GFS Chemicals – Veritas Double Distilled), nitric (99.999% trace metals basis, Sigma-Aldrich), and sulfuric (99.999% trace metals basis, Alfa Aesar). We prepared transfer solutions of all three acids of an intermediate concentration between the final (0.1 M) and stock in a container that was thoroughly cleaned with piranha solution (subsequently rinsed 20 times with the working acid and Millipore water from the dispenser) to avoid adventitious organic contamination that can lead to irreproducibility and poor performance. To prepare the electrolyte for an experiment, a fixed mass of this transfer solution was dispensed by pouring it directly into the cell on a mass balance and a fixed mass of Millipore water, to dilute to 0.1 M, was then added directly from the dispenser, thus entirely removing the potential of organic contamination from volumetric glassware. Our piranha cleaning and electrolyte preparation protocols were crucial and ensured stable performance between multiple trials and polishing steps.

### Electrochemical analysis

All glassware was piranha-cleaned and subsequently rinsed at least 20 times with Millipore water to remove sulfates from the piranha solution and stored with Millipore water. Electrochemical measurements with an RDE setup were performed in a three-electrode glass cell with a rotator (Pine Research Instruments) and potentiostat (BioLogic VMP-300). This cell was cleaned with aqua regia and subsequently with piranha solution, separately, to remove any metal contamination and organic contamination that has been known to decrease measured activity on Pt^37^. To avoid non-Pt contamination all three electrodes were composed of pure Pt. The working electrode consisted of the aforementioned Pt disk placed in a Pine Research Instruments Teflon ChangeDisk RDE holder and shaft assembly. The counter electrode was a Pt wire cleaned and flame annealed prior to measurements (stored in 30 wt% HNO_3_). The reference electrode was a constructed reversible hydrogen electrode (RHE) consisting of two gas dispersion tubes of different diameters with the smaller tube on the inside supplying hydrogen gas. A clean and flame annealed Pt wire (stored in 30 wt% HNO_3_), with a coiled end to increase surface area, was placed between the inner and outer dispersion tube walls and the assembly was submerged in the electrolyte being tested. We report fully uncompensated resistance corrected (*R*_u_, measured by potential impedance spectroscopy (PEIS) after electrode conditioning) potentials vs. RHE after further calibrating our in-house RHE to the 0 V vs. RHE definition as measured by the crossover point of HER/HOR on the Pt disk; the electrode was no more than 0.1 mV off in all trials. We performed the following CV electrochemical experiments, in triplicate (separate electrolyte batch and separate piranha cleaning for each electrolyte species) in order in gas saturated electrolyte: Pt disk electrode conditioning (100 cycles @ 500 mV s^–1^ in N_2_, from 0.025 V_RHE_ to 1.4 V_RHE_ with start and end points of 0.4 V_RHE_ and 0.025 V_RHE_ respectively), PEIS, H_UPD_ ECSA estimation (3 cycles @ 500 mV s^–1^ in N_2_, from 0.025 V_RHE_ to 1.0 V_RHE_ with start and end points of 0.4 V_RHE_ and 0.025 V_RHE_ respectively), ORR O_2_ (3 cycles @ 20 mV s^–1^ in O_2_, 1 V_RHE_ to –0.01 V_RHE_), polarization in N_2_ for ORR correction (2 cycles @ 20 mV s^–1^ in N_2_, 1 V_RHE_ to –0.01 V_RHE_), HER/HOR (3 cycles @ 20 mV s^–1^ in H_2_, –0.1 V_RHE_ to 0.1 V_RHE_), and OER (5 cycles, 1.2 V_RHE_ to 1.7 V_RHE_ @ 20 mV s^–1^ in O_2_), all at 1600 rpm. Before triplicate measurements, we performed the electrochemical testing series outlined above, once, which allowed trace sulfates from piranha cleaning to be removed. The Pt disk was rinsed with Millipore water (*R* = 18 MΩ cm) directly from the dispenser immediately after removing it from electrolyte, dried with N_2_, and then immediately taken (in air) for the physical characterization (XPS and AFM) measurements described below.

For CVs reported for the ORR, HER, and HOR, the average of triplicate measurements of the third cycle is displayed in Fig. 2 as these traces overlap almost exactly. However, for the OER, activity decreased with cycles (Supplementary Fig. 3), so the second cycle is used to represent performance as the likely most reduced Pt surface without capacitive effects that are present in the first cycle. As it is typical in OER literature^67^, we report the average of the anodic and cathodic sweeps, though the raw data from this measurement is available in Supplementary Fig. 3.

### Physical characterization

XPS was performed using a PHI III Versaprobe instrument with an Al Kα (1486 eV) source employing a 224 and 55 eV pass energies to collect Survey (Su) and high resolution (HR) spectra, respectively. All measurements were collected using the instrument’s neutralizer and the Ar^+^ gun in neutralizing mode on a 100 μm × 100 μm high power spot (100 W, 20  kV) at both the center and edge of the Pt disk and representative spectra are shown in this work. The sample-to-detector angle was 45° (default) and the X-ray beam was perpendicular to the sample with the stage height being optimized for maximal signal intensity. A Park Systems XE-70 atomic force microscope equipped with a pre-mounted Mikromasch NSC15/Al BS tip was used to obtain 10 μm × 10 μm size non-contact images of the surface with topographical information (i.e. roughness factor) being calculated in Gwyddion^93^ software. We define the roughness factor (RF) as the true (as measured with AFM) surface area divided by the geometric surface area. To confirm this normalization procedure, H_UPD_ measurements were performed to estimate the surface area and are presented in Supplementary Fig. 2.

### Chemical/molecular analysis

The electrolyte collected after electrochemical testing of the Pt disk in 0.1 M HNO_3_ was analyzed by ^1^H NMR for the presence of ammonia as a potential side-product if Pt electrocatalyzed the reduction of nitrate (${{{{{\rm{N}}}}}}{{{{{{\rm{O}}}}}}}_{3}^{-}$). Samples, including calibration standards from NH_4_OH (28% in water, 99.99% metals basis, Sigma Aldrich), were prepared for analysis by adding 500 μL of analyte to a 20 mL scintillation vial, followed by stepwise addition and mixing of 50 μL D_2_O (99.9 atom % D, Acros Organics) as a locking agent and 100 μL dilute (845 μM) CHCl_3_ (Certified ACS Reagent Grade, >99.8%, Fisher Chemical) as an internal standard. Acidification to stabilize NH_4_^+^ in solution was unnecessary given the acidic nature of the 0.1 M HNO_3_ electrolyte employed. Spectra were recorded on a Varian Inova NMR spectrometer operating at a frequency of 600 MHz at 25 °C with a 5 mm triple resonance pulsed-field gradient probe using a selective pulse gradient spin echo sequence adapted from literature^78^. Briefly, this sequence consisted of a 90° pulse followed by a selective 180° gradient echo pulse. The number of scans ranged from 96 scans (10-min scan time) for calibration standards at concentrations from 25 to 100 μM to 192 scans (20-min scan time) for calibration standards from 5 to 10 μM and for test analytes. Calibration data was collected in triplicate for solutions ranging in known concentration from 5 to 100 μM to generate a plot of NH_4_^+^:CHCl_3_ integral ratios. This plot was subsequently fit via linear regression to establish an equation for quantification, and while 5 μM was found to be the limit of detection after 192 scans, a low signal-to-noise ratio achieved at this concentration with the given scan parameters precluded its addition to the calibration range, therefore 10 μM was taken as the lower limit of quantification.

### Computational details

Periodic spin−polarized DFT calculations were performed using the Vienna Ab−initio Simulation Package (VASP version 5.4.4)^94^ with the RPBE exchange correlation functional^95^, a plane-wave basis set with a cutoff kinetic energy of 500/400 eV for metal/metal oxide surfaces, and the projector-augmented wave (PAW) method^96^. PAW pseudo-potentials were selected according to the Materials Project (MP) database^97^. The Pt metal surface was modeled as a five−layer p(3 × 3) Pt(111) fcc slab with RPBE optimized lattice constant of 3.99 Å, in agreement with previously reported values^98^. We further modeled a Pt(111) surface covered with 1 ML H* on fcc hollow sites relevant for HER and used both RPBE and BEEF-vdW functionals^91^ to evaluate the anion adsorption energies. The BEEF-vdW functional provides a reasonable description of van der Waals dispersion interactions while maintaining an accurate prediction of chemisorption energies^91^. The oxidized Pt surface at high potentials relevant for OER was modeled as a *P*4_2_/*mnm* [136] PtO_2_(110) surface. Specifically, we used a c(1 × 2) surface cell with two distinct bridging O atoms (O_br_) and two distinct fivefold coordinated Pt atoms (5c-Pt) and consisted of four stoichiometric PtO_2_ layers. The Brillouin zone was sampled with a Γ−centered (3 × 3 × 1) Monkhorst−Pack grid^99^. In all of the slabs, the top two layers and adsorbed species were fully relaxed, whereas the bottom layers were constrained at the bulk positions. The slabs were separated in the perpendicular z-direction by at least 15 Å of vacuum, and a dipole correction was applied. The electronic and force convergence criterion were 10^−5^/10^−4^ eV for metals/metal oxides and 0.05 eV Å^−1^, respectively. Solvation at the metal surface were modeled using the implicit solvation method implemented in VASPsol^100^ with a dielectric constant of 80 and a Debye screening length of 3 Å. The nonelectrostatic coefficient was set to zero to avoid numerical instabilities in the electrolyte region.

To identify the role of acid electrolyte anions on electrocatalytic performance on Pt(111), we evaluated the adsorption of anions (A^*n*−^, *n* = 1, 2)^16,22^. For each anion, we considered several configurations of anion adsorption geometries on Pt(111) surface and the most stable adsorption configuration is shown in Fig. 3a. The optimal adsorption of ${{{{{\rm{Cl}}}}}}{{{{{{\rm{O}}}}}}}_{4}^{-}$, ${{{{{\rm{HS}}}}}}{{{{{{\rm{O}}}}}}}_{4}^{-}$, and ${{{{{\rm{S}}}}}}{{{{{{\rm{O}}}}}}}_{4}^{2-}$ to the Pt(111) surface occurs with three O atoms on top sites, with the Cl/S atoms located above the hollow position and the final O atom positioned through the Cl–/S–O bond perpendicular to the surface. For ${{{{{\rm{HS}}}}}}{{{{{{\rm{O}}}}}}}_{4}^{-}$, the H atom is connected through the O atom in the S–O bond perpendicular to the surface. In the most stable geometry for ${{{{{\rm{N}}}}}}{{{{{{\rm{O}}}}}}}_{3}^{-}$, two O atoms adsorb on top sites and the third O atom is located through the N–O bond perpendicular to the surface. The adsorption free energy *(*Δ*G*_adsorption_(A^*n*−^)) of the anions from solution was calculated as,

1 $$\Delta {G}_{{{{{{\rm{adsorption}}}}}}}({{{{{{\rm{A}}}}}}}^{n-})=	\,\Delta {{G}}_{{{{{{\rm{CHE}}}}}}}({{U}}_{{{{{{\rm{RHE}}}}}}})-\Delta {{G}}_{{{{{{\rm{solvation}}}}}}}({{{\mathrm{H}}}}_{n}{{{\mathrm{A}}}}({\rm g}))\\ 	-\Delta {G}_{{{{{{\rm{dilution}}}}}}}({{{{{{\rm{H}}}}}}}_{n}{{{{{\rm{A}}}}}}({{{{{\rm{solvated}}}}}}))$$

where Δ*G*_solvation_(H_*n*_A(g)) and Δ*G*_dilution_(H_*n*_A(solvated)) are the solvation free energy and dilution free energy calculated using experimental literature data for standard thermodynamic relations (see Supplementary Table 3 for more details)^101,102^. The effect of the electrode potential on the Δ*G*_adsorption_(A^*n*−^) was determined by Δ*G*_CHE_(*U*_RHE_) term, which was calculated from DFT and computational hydrogen electrode (CHE)^66^.

2 $$\Delta {G}_{{{{{\rm{CHE}}}}}}({U}_{{{{{\rm{RHE}}}}}})=\Delta {G}_{{{{{\rm{CHE}}}}}}({U}_{{{{{\rm{RHE}}}}}}=0{{{\rm{V}}}})-ne{U}_{{{{{\rm{RHE}}}}}}$$

where *n*, *e*, and *U*_RHE_ are the number of electrons involved in the reaction, the elementary charge, and the electrode potential with respect to the reversible hydrogen electrode (RHE), respectively. The adsorption free energies of the anions at *U*_RHE_ = 0 V and standard conditions were calculated as Δ*G*_CHE_(*U*_RHE_ = 0 V) = Δ*E*_DFT_ + Δ*E*_ZPE_ + ${\int }_{0}^{298.15}{C}_{{{{{{\rm{p}}}}}}}{{{{{\rm{d}}}}}}T$ − TΔ*S*, where Δ*E*_DFT_ is the difference in DFT calculated electronic energy, Δ*E*_ZPE_ is the difference in zero-point energies, ${\int }_{0}^{298.15}{C}_{{{{{{\rm{p}}}}}}}{{{{{\rm{d}}}}}}T$ is the difference in integrated heat capacity from 0 to 298.15 K, Δ*S* is the change in entropy of the adsorbed species, and calculated using the harmonic oscillator approximation as implemented in the ASE with respect to the catalyst surface, H_*n*_A(g) and H_2_(g). For gas molecules, the ideal gas approximation with experimental molecular data^103^ and a partial pressure of 101,325 Pa was employed except for H_2_O, for which a partial pressure of 3534 Pa corresponding to the vapor pressure of H_2_O was used.

Both for the ORR and OER, we considered the associative reaction mechanism consisting of four proton-coupled electron transfer (PCET) reactions^66,104^. To compare the competitive adsorption of anions and ORR/OER intermediates, the adsorption free energies of *OOH, *O, and *OH intermediates were calculated. It is important to note that ab initio molecular dynamics (AIMD) simulations of acid anion electrolytes in contact with the surfaces with explicit water molecules would be needed to accurately model the system and are beyond the scope of this study. The coverage of anions and ORR/OER reaction intermediates could potentially influence the adsorption free energies^105^, however, a detailed study including adsorbate−adsorbate interactions also is beyond the scope of this study. Finally, to assess the electronic structure of the Pt surfaces, we computed the Pt-projected density of states (PDOS) on Pt(111), H*-covered Pt(111), and PtO_2_(110) surfaces (Supplementary Fig. 8) and did not observe significant changes of PDOS in the H*-covered Pt(111) compared to the pristine Pt(111) surface.

## Supplementary information

Supplementary Information

## Data Availability

Source data are provided with this paper. All raw data plotted in this work can also be accessed on figshare.com through 10.6084/m9.figshare.16679665. The optimized DFT structures are available in the data repository https://data.dtu.dk/, and can be accessed using the link 10.11583/DTU.18277970.
