# Supplementary material for: Acid anion electrolyte effects on platinum for oxygen and hydrogen electrocatalysis
Source: Commun Chem. 2022 Feb 18;5:20. doi: 10.1038/s42004-022-00635-1 (PMC9814610; doi:10.1038/s42004-022-00635-1)
Supplement: Supplementary file 1 — Supplementary Information [file 42004_2022_635_MOESM1_ESM.pdf]

Supplementary Information

Acid Anion Electrolyte Effects on Platinum for Oxygen and Hydrogen Electrocatalysis

Gaurav Ashish Kamat[†,a,b], José A. Zamora Zeledón[†,a,b], G. T. Kasun Kalhara Gunasooriya[c], Samuel M. Dull[a,b], Joseph T. Perryman[a,b], Jens K. Nørskov[c], Michaela Burke Stevens[a,b,*] and Thomas F. Jaramillo[a,b,*]

a. Department of Chemical Engineering, Stanford University, 443 Via Ortega, Stanford, California 94305, United States
b. SUNCAT Center for Interface Science and Catalysis, SLAC National Accelerator Laboratory, 2575 Sand Hill Road, Menlo Park, California 94025, United States
c. Catalysis Theory Center, Department of Physics, Technical University of Denmark, 2800 Kongens Lyngby, Denmark

† These authors contributed equally

* Corresponding authors: mburkes@stanford.edu and jaramillo@stanford.edu.

Table of Contents

Supplementary Figure 1: Physical characterization. 3

Supplementary Figure 2: Conditioning and H_{UPD} in N_2 4

Supplementary Figure 3: HNO_3 electrolyte NMR. 5

Supplementary Figure 4: OER cycling. 6

Supplementary Figure 5: Anion adsorption modeling on $PtO_2(110)$ surface. 7

Supplementary Figure 6: Anion adsorption modeling on H*-covered Pt(111) 7

Supplementary Figure 7: Computational Pourbaix diagram of Pt 8

Supplementary Figure 8: Pt-Projected DOS on (a) Pt(111), H*-covered Pt(111), and (b) $PtO_2(110)$ surfaces 9

Supplementary Table 1: HER/HOR/ORR/OER performance metrics. 10

Supplementary Table 2: Electrolyte anions. 11

Supplementary Table 3: DFT-calculated adsorption, solvation, & dilution free energies. . 11

Supplementary Table 4: Comparison of DFT anion adsorption free energies on Pt(111) and $PtO_2(110)$ at 1.6 V_{RHE} 11

Supplementary Table 5: DFT-calculated anion adsorption on H*-covered Pt(111). 12

Supplementary Table 6: DFT-calculated bond lengths (Å) on Pt(111) and H*-covered Pt(111). 13

Supplementary Table 7: DFT-calculated Bader charge analysis of anions on H*-covered Pt(111). 14

Supplementary Note 1 14

Supplementary References 17

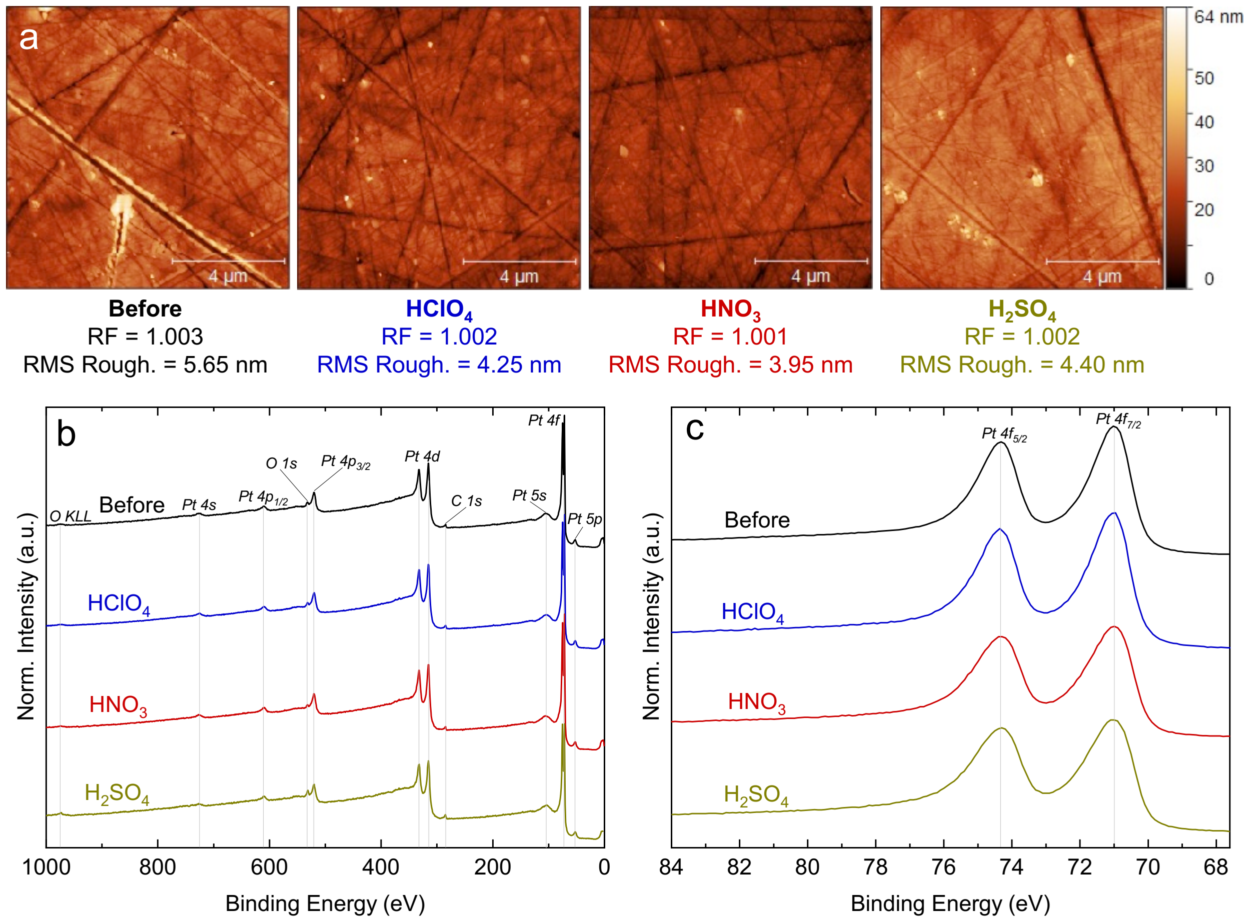

Supplementary Figure 1: Physical characterization. (**a**) Representative AFM topography images with overlayed RF and RMSR values, (**b**) representative survey XPS, and (**c**) representative high resolution XPS of the Pt disk before and after electrochemical testing in each electrolyte. XPS measurements of the clean, polished, flame-annealed Pt disk were taken before and then immediately after electrochemical testing in each electrolyte; all spectra collected at various points on the surface look exactly the same, and therefore we only show one set of spectra for conciseness.

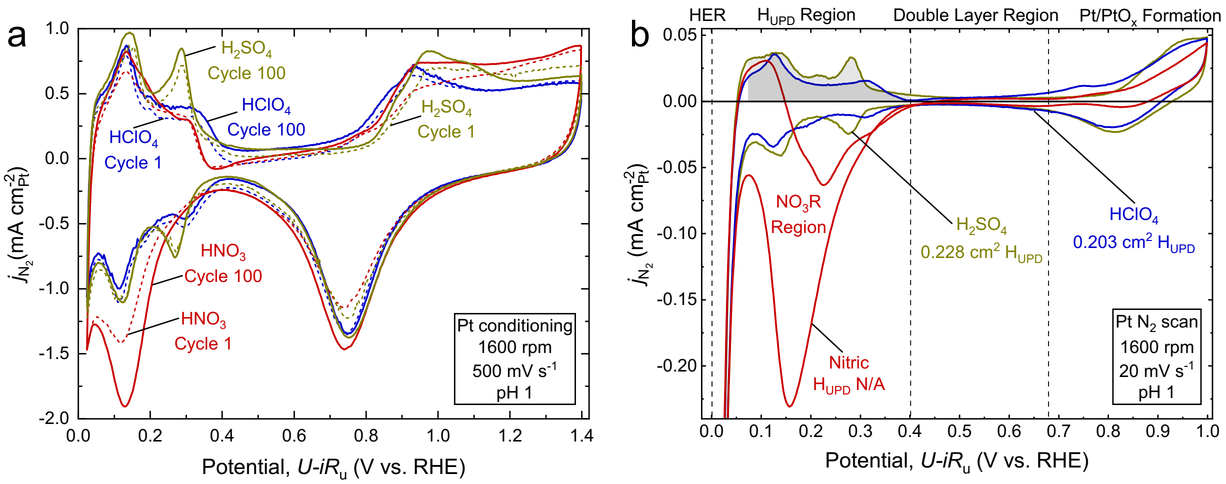

Supplementary Figure 2: Conditioning and H_{UPD} in N_2. (**a**) Electrode conditioning[1] (under N_2-saturation) in each electrolyte. (**b**) N_2 H_{UPD} CVs in each electrolyte with estimated areas of ~0.203 cm^2_{Pt} in $HClO_4$ and ~0.228 cm^2_{Pt} in H_2SO_4.[1] H_{UPD} area estimation is not possible in HNO_3 due to the NO_3R region being convoluted with hydrogen adsorption and desorption. While the nominal shape of the H_{UPD} region is impacted by the electrolyte composition, the H_{UPD}-estimated catalyst surface areas in $HClO_4$ and H_2SO_4 are in rough agreement with those from (more precise and accurate) AFM. It's important to note that while it is a standard Pt ECSA method,[1] H_{UPD} ECSA is not as numerically exact as AFM due to the facet dependence of the specific charge transfer.[1-6] Since we do not employ a Pt single crystal, we used the standard theoretical specific charge transfer value of 210 μC cm^{-2}_{Pt}, which averages this material property based on the theoretically dominant facets of Pt.[1-6] Ultimately, any *in situ* ECSA methods require control measurements on flat smooth surfaces correlated to the AFM surface area to empirically determine specific material properties such as specific charge transfer (for UPD methods) or specific capacitance (for scan rate — double layer capacitance — methods).[1-6]

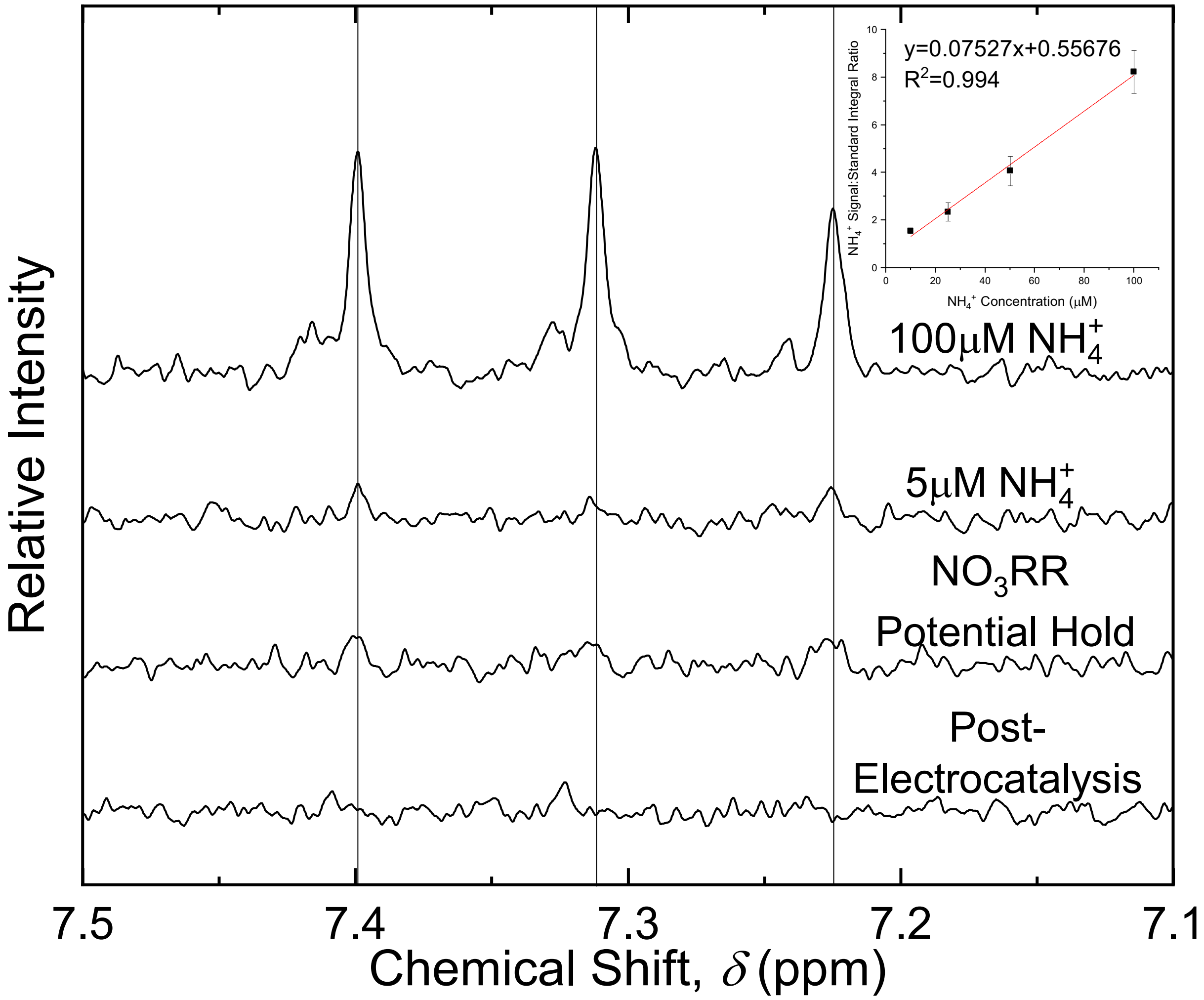

Supplementary Figure 3: HNO_3 electrolyte NMR. Representative ^{1}H NMR spectra of 100 μM and 5 μM NH_4OH calibration standards with representative spectra of 0.1 M HNO_3 electrolyte post-electrochemistry (N_2 CVs + HOR/HER/OER/ORR) and after potential hold at 0.16 V_{RHE} for 10 minutes (attempting to accumulate NO_3RR products), indicating that no quantifiable concentration of NH_4^+ (and consequently no NH_3) was present in either solution, and therefore that any potential NO_3RR during testing had negligible effects on electrolyte composition. Notably, at concentrations above 50 μM, side bands are observable at +0.015 ppm which originate from deuterium exchange between D_2O and NH_4^+ to yield NH_3D^+. The top-right inset shows the fitted calibration curve for standard solutions from 10-100 μM where error bars on points represent a single standard deviation following triplicate scans.

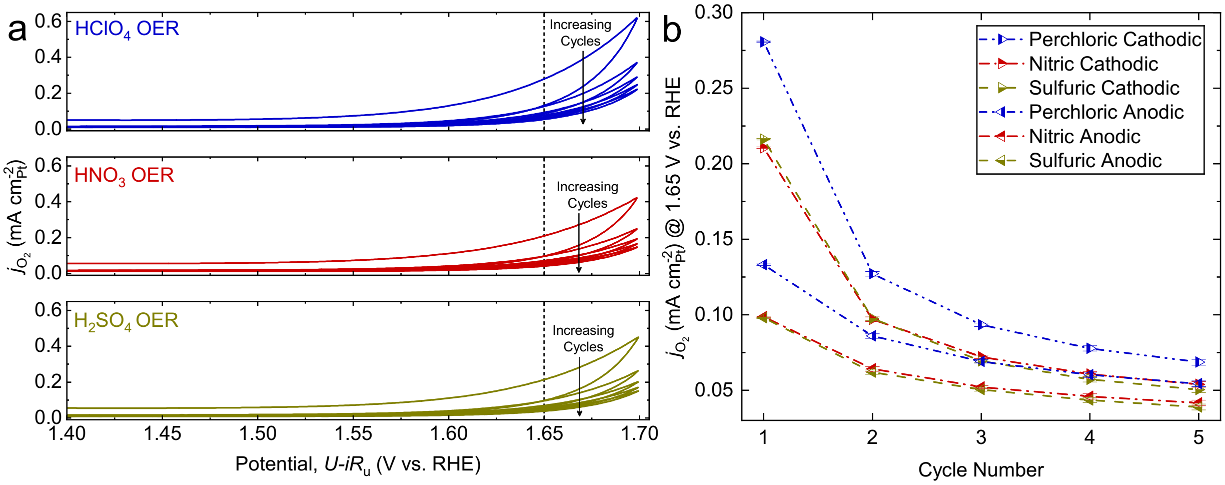

Supplementary Figure 4: OER cycling. (**a**) Overlaid OER CV cycles in each electrolyte indicating the gradual decrease in activity with increasing cycles. (**b**) Cathodic and anodic sweep current density at 1.65 V vs. RHE (dashed line in (**a**)) for the OER as a function of cycle. Error bars in (**b**) are the standard deviation from triplicate measurements in three separate electrolyte batches.

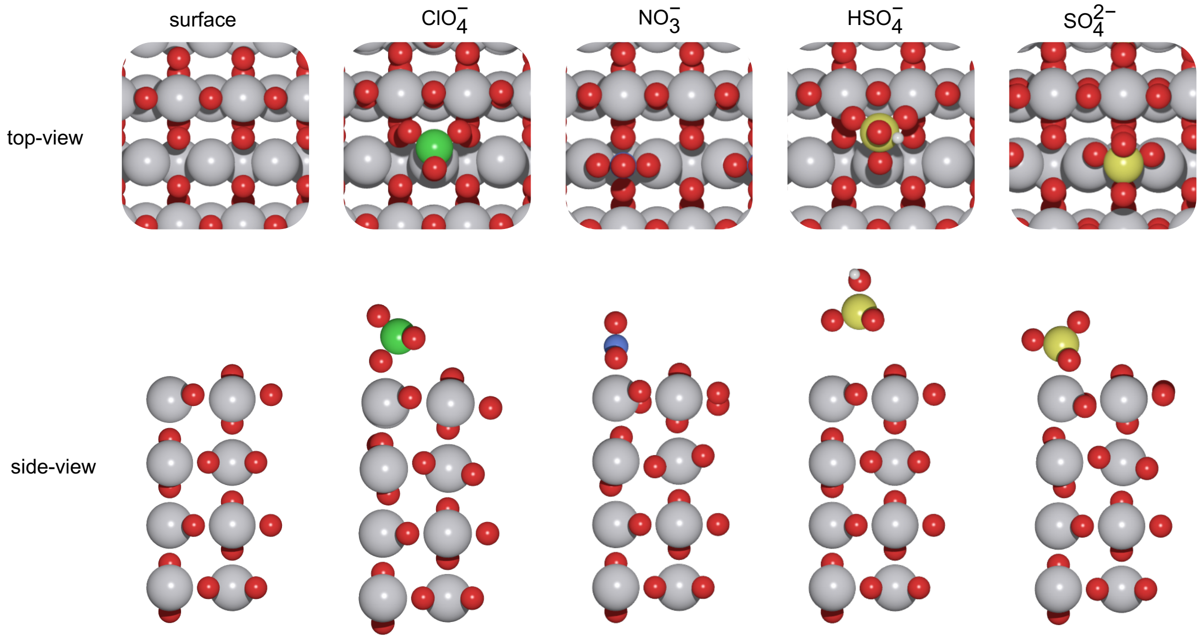

Supplementary Figure 5: Anion adsorption modeling on PtO_2(110) surface. The most stable adsorption configurations of anions on PtO_2(110) surface. Color coded: O: red, H: white, Cl: green, N: blue, and S: yellow.

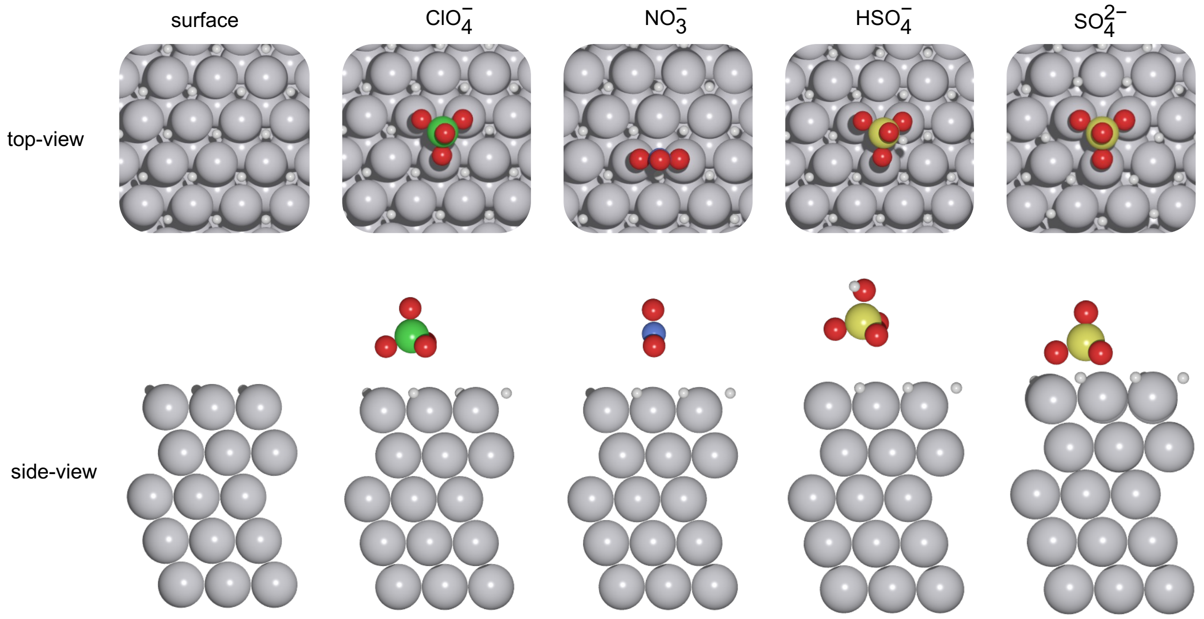

Supplementary Figure 6: Anion adsorption modeling on H*-covered Pt(111). The most stable adsorption configurations of anions on Pt(111) surface with 1 ML coverage of adsorbed H*. Color coded: O: red, H: white, Cl: green, N: blue, and S: yellow.

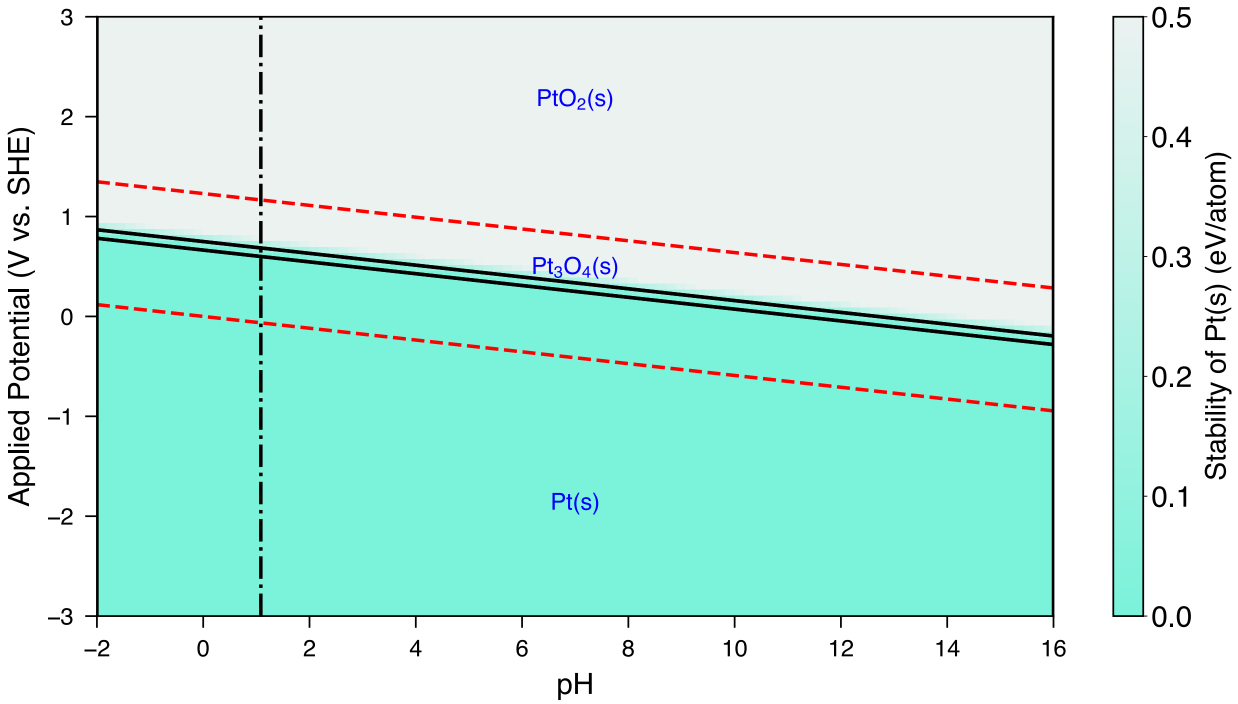

Supplementary Figure 7: Computational Pourbaix diagram of Pt.[7,8] Pourbaix diagram was constructed with aqueous ion concentrations 10^{-6} M at 25 °C for the Pt−H_2O phases. Color bar on the right indicates the relative stability of the Pt(s) (green = more stable, gray = less stable). Dashed red lines are the equilibrium potentials for oxygen ($E_{O2/H2O}$ = 1.23 V_{RHE}) and hydrogen ($E_{H+/H2}$ = 0.00 V_{RHE}) electrochemistry. Dashed black vertical line corresponds to pH = 1.

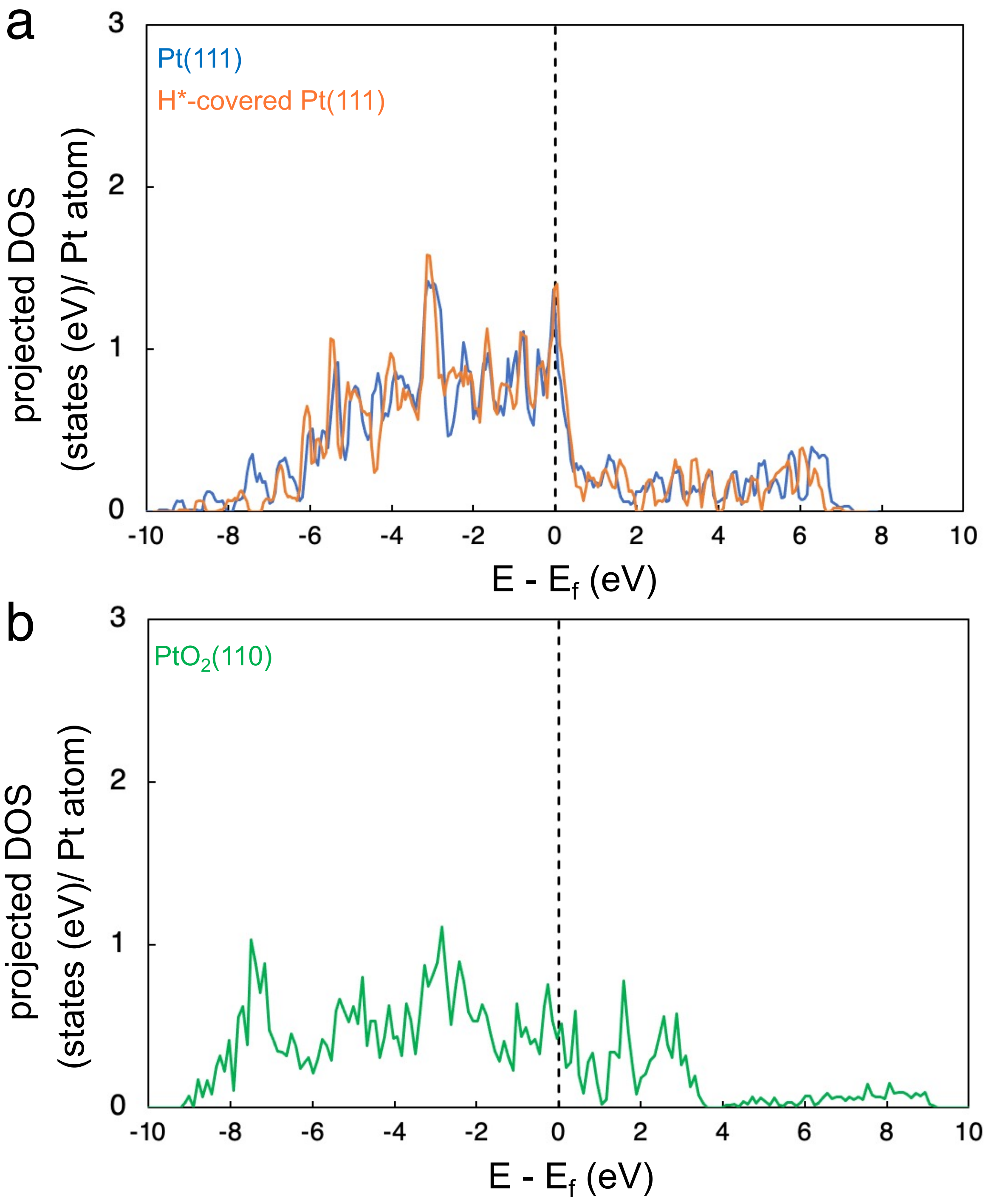

Supplementary Figure 8: **Pt-Projected DOS on (a) Pt(111), H*-covered Pt(111), and (b) PtO_2(110) surfaces**.

Supplementary Table 1: HER/HOR/ORR/OER performance metrics. Average hydrogen and oxygen electrocatalysis performance metrics with corresponding standard deviations (error) from triplicate measurements (averaging the cathodic and anodic CV scans). Tabulated data is shown based on the 3rd CV cycle for HER/HOR/ORR and the 2nd CV cycle for OER. Within each table-cell, numbers correspond to $HClO_4$ (top, blue), HNO_3 (middle, red), H_2SO_4 (bottom, yellow). Kinetic current densities (j_k) for HER estimated based on the HOR mass transport limited current densities; the high asymmetry between ORR and OER makes such estimations for OER less accurate and therefore are not shown here. To calculate exchange current density, we extrapolate the linear fit used to calculate the Tafel slope (Potential vs. $\log_{10} j$) to equilibrium potential (0V_{RHE} for HER/HOR, 1.229V_{RHE} for ORR/OER) where j represents j_k for HOR and ORR, j_{H_2} for HER, and j_{O_2} for OER. Average (Avg.) turnover frequency (TOF) for ORR and OER assumes full $4e^-$ selectivity. HER and HOR Avg. TOF are derived from their kinetic current densities according to the HOR mass transport limited current densities.

	HER		HOR		ORR		OER	
	Value	Error	Value	Error	Value	Error	Value	Error
Onset Potential (V_{RHE}) @ 0.1 mA cm_{Pt}^{-2}	N/A	N/A	0.0014<br>0.0012<br>0.0008	1.9E-4<br>3.4E-5<br>5.3E-5	N/A	N/A	1.64<br>1.65<br>1.65	9.4E-4<br>2.7E-4<br>3.5E-4
Onset Potential (V_{RHE}) @ –0.1 mA cm_{Pt}^{-2}	-0.0012<br>-0.0011<br>-0.0011	1.7E-4<br>4.7E-5<br>4.5E-5	N/A	N/A	0.980<br>0.969<br>0.935	8.8E-4<br>8.6E-4<br>7.6E-4	N/A	N/A
Tafel Slope (mV dec^{-1})	26.9<br>25.4<br>27.4	0.6<br>0.9<br>0.9	21.9<br>19.8<br>21.6	0.9<br>0.4<br>1.0	61.1<br>62.4<br>61.2	0.4<br>0.7<br>0.5	125.1<br>157.9<br>166.4	1.1<br>1.4<br>1.8
Exchange Current Density (mA cm_{Pt}^{-2})	1.0171<br>1.0131<br>1.0170	0.0003<br>0.0004<br>0.0005	1.0107<br>1.0063<br>1.0109	0.0007<br>0.0003<br>0.0006	1.70E-6<br>1.43E-6<br>5.86E-7	1.94E-7<br>2.57E-7<br>8.24E-8	4.21E-5<br>1.56E-4<br>2.16E-4	2.49E-6<br>6.63E-6<br>5.48E-5
Overpotential, η, (V) @ –10 mA cm_{Pt}^{-2}	-0.020<br>-0.020<br>-0.020	9.6E-6<br>7.5E-6<br>8.1E-6	N/A	N/A	N/A	N/A	N/A	N/A
Avg. TOF (s^{-1}) @ η = 10 mV (HER/HOR), 0.9 V_{RHE} (ORR), and η = 420 mV (OER)	0.071<br>0.072<br>0.073	0.001<br>0.001<br>0.001	0.096<br>0.093<br>0.083	0.001<br>0.001<br>0.001	0.12<br>0.058<br>0.016	1.5E-3<br>7.0E-4<br>1.7E-4	0.132<br>0.098<br>0.102	1.3E-3<br>1.1E-3<br>1.2E-3
Kinetic Current Density (mA cm_{Pt}^{-2}) @ η = 10 mV (HER/HOR), and 0.9 V vs. RHE (ORR)	-3.35<br>-3.40<br>-3.46	0.06<br>0.05<br>0.06	4.54<br>4.43<br>3.92	0.06<br>0.06<br>0.07	-2.52<br>-1.37<br>-0.35	0.04<br>0.02<br>0.00	N/A	N/A

Supplementary Table 2: Electrolyte anions. Acid electrolytes, corresponding pK_a values for acids, main corresponding anion (A^{n-}), and the competing anion species considered in this study.

Acid electrolytes	pK_a	A^{n-}	Competing anion species
$HClO_4$	< 0	ClO_4^-	–
HNO_3	< 0	NO_3^-	–
H_2SO_4	< 0	HSO_4^-	SO_4^{2-}

Supplementary Table 3: DFT-calculated adsorption, solvation, & dilution free energies. DFT calculated adsorption free energy $\Delta G_{CHE}(U_{RHE} = 0\ V)$ on Pt(111) surface, solvation free energy ($\Delta G_{solvation}(H_nA(g))$), and dilution free energy ($G_{dilution}(H_nA(solvated))$). The solvation free energy and dilution free energy values are calculated using experimental literature data for standard thermodynamic relations.[9,10]

A^{n-}	$\Delta G_{CHE}(U_{RHE} = 0\ V)$	$\Delta G_{solvation}(H_nA(g))$	$\Delta G_{dilution}(H_nA(solvated))$
ClO_4^-	1.78	-0.96	-0.13
NO_3^-	0.96	-0.32	-0.13
HSO_4^-	1.45	-0.68	-0.13
SO_4^{2-}	1.71	-0.57	-0.19

Supplementary Table 4: Comparison of DFT anion adsorption free energies on Pt(111) and PtO_2(110) at 1.6 V_{RHE}. DFT calculated adsorption free energies of anions [$\Delta G_{adsorption}(A^{n-})$] on the Pt(111) and PtO_2(110) surfaces at 1.6 V vs. RHE corresponding to OER conditions.

A^{n-}	Pt(111)	PtO_2(110)
ClO_4^-	0.18	0.37
NO_3^-	-0.64	-0.78
HSO_4^-	-0.54	0.37
SO_4^{2-}	-1.66	-0.87

Supplementary Table 5: DFT-calculated anion adsorption on H*-covered Pt(111). DFT calculated adsorption free energies of anions [$\Delta G_{\mathrm{adsorption}}(A^{n-})$] on the Pt(111) and 1 ML H*-Pt(111) surfaces at 0 V vs. RHE corresponding to HER conditions.

A^{n-}	Pt(111)	1ML H*-Pt(111)	
	RPBE (eV)	RPBE (eV)	BEEF-vdW (eV)
ClO_4^-	1.78	2.44	1.95
NO_3^-	0.96	1.88	1.58
HSO_4^-	1.06	1.90	1.44
SO_4^{2-}	1.54	2.64	1.92

Supplementary Table 6: DFT-calculated bond lengths (Å) on Pt(111) and H*-covered Pt(111). DFT-calculated bond lengths (Å) of nearest surface Pt atom and anion O atom on Pt(111) and H*-covered Pt(111) surfaces.

A^{n-}		Pt(111) RPBE	1ML H*-Pt(111) RPBE	1ML H*-Pt(111) BEEF-vdW
ClO_4^-				
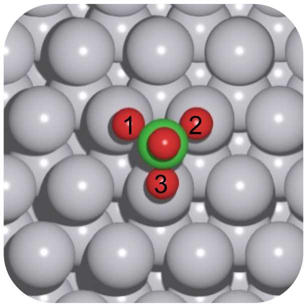	Pt-O_1	2.56	3.58	3.31
	Pt-O_2	2.31	3.36	3.04
	Pt-O_3	2.31	3.35	3.04
NO_3^-				
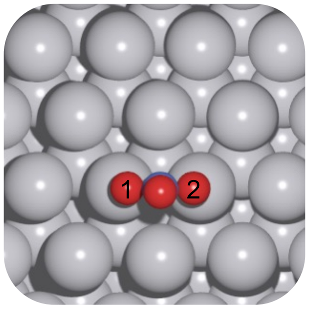	Pt-O_1	2.16	3.43	3.35
	Pt-O_2	2.16	3.40	3.32
HSO_4^-				
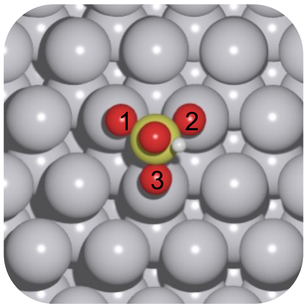	Pt-O_1	2.40	4.19	3.24
	Pt-O_2	2.33	3.58	2.78
	Pt-O_3	2.38	3.91	3.18
SO_4^{2-}				
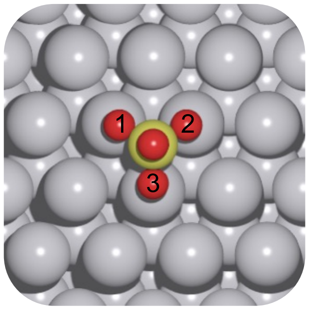	Pt-O_1	2.11	2.25	2.25
	Pt-O_2	2.11	2.25	2.25
	Pt-O_3	2.12	2.26	2.26

Supplementary Table 7: DFT-calculated Bader charge analysis of anions on H*-covered Pt(111). The charge of the anions on H*-covered Pt(111) have been referenced to the charge of the anions on Pt(111).

A^{n-}	1ML H*-Pt(111) RPBE
ClO_4^-	0.17
NO_3^-	0.01
HSO_4^-	-0.03
SO_4^{2-}	-0.08

Supplementary Note 1

We performed physical characterization measurements before and after the electrochemical experiments (**Supplementary Fig. 1**) as described in the Methods section. Key to our discussion and hypotheses is the analysis of the active surface as electrocatalysis is known to affect the morphology of surfaces. Specifically in this study, the possibility of anions inducing a surface reaction that creates a new chemical species (e.g. $PtSO_4$) cannot be discounted so we employed XPS and AFM to understand the chemical composition and surface topography, respectively, of the Pt disk after electrochemistry. **Supplementary Fig. 1a** displays the survey XPS and inset high resolution spectra of the Pt 4*f* region with all peaks assigned in all three electrolytes. Other than adventitious carbon and corresponding oxygen, there appears to be no significant contamination or oxidation of the Pt near-surface with a constant Pt $4f_{7/2}$ peak position at 71 eV and a characteristic asymmetric shape of the Pt 4*f* peaks.[11] Therefore, no appearance of another chemical species resulting from reaction of an electrolyte species is observed and therefore such phenomena is not affecting the activity measurements. It should be noted that the OER onset region is well above the potential at which oxidation of the Pt surface begins, though we did not see any Pt oxides with XPS. **Supplementary Fig. 1b** shows the AFM images of the surface before/after electrochemistry and the low roughness factor that was achieved with a meticulous alumina polishing procedure. As expected for mechanical polishing, there are minor nano-scratches visible

but this has been previously reported[1] to not interfere with measured activity and our AFM measurements verify these features do not significantly increase the exposed surface area. We note that the images acquired after electrochemistry do not differ significantly in qualitative appearance or quantitative topographical characteristics (roughness factor, RMS roughness) from those taken before electrochemistry, implying that the measured electrochemical performance shifts are not due to material surface area changes.

The current response to the electrode conditioning protocol (100 cycles, 0.025 V_{RHE} to 1.4 V_{RHE} @ 500 mV sec^{-1} in N_2) is plotted in **Supplementary Fig. 2a** and highlights certain surface processes relevant to preparing/conditioning the Pt disk for electrochemical measurements.[1] Although collected at different scan rates, 500 and 20 mV sec^{-1} respectively, **Supplementary Fig. 2a** is generally consistent with the features described in the main text for **Figure 1**. In **Supplementary Fig. 2a,** for each of the three electrolytes, there are clear changes between the first cycle and last cycle H_{UPD} features as the peaks gain greater definition as the Pt surface restructures into the most favorable *in situ* structure, which in addition to piranha cleaning, is also needed to archive the reference expected state-of-the-art ORR performance of Pt.[1] Notably, the H_{UPD} oxidation features in in **Supplementary Fig. 2a** are better defined than in **Figure 1**.The region between 0.6 V_{RHE} and 1.3 V_{RHE} shows Pt oxidation and reduction, which is thought to be essential in conditioning the surface for the most optimal electrocatalysis on Pt.[1] Additionally, a non-horizontal slope of the region around 1.3 V_{RHE} in the anodic scan has been shown to indicate carbon contamination in perchloric acid whereas a flat slope implies a clean surface.[1] Extending this to the other acids, it appear that the anodic scan in all 3 electrolytes reach a plateau around 1.2–1.4 V_{RHE}, suggesting no significant organic contamination during testing. However, while

perchloric acid and sulfuric acid have relatively flat profiles in that region, nitric acid appears to have the onset of another redox feature above 1.3 V_{RHE} as the region did not significantly change after 100 cycles. Overall, extensive physical and chemical characterization of the Pt disk show it to remain highly reduced *ex situ*, clean of contamination during reaction, without the formation of other chemical phases during reaction, and demonstrates no significant changes to surface topography due to electrochemical testing.

Supplementary References

1. Shinozaki, K., Zack, J. W., Richards, R. M., Pivovar, B. S. & Kocha, S. S. Oxygen Reduction Reaction Measurements on Platinum Electrocatalysts Utilizing Rotating Disk Electrode Technique. *J. Electrochem. Soc.* **162 (10)**, F1144-F1158 (2015).
2. Zamora Zeledón, J. A. *et al.* Probing the Effects of Acid Electrolyte Anions on Electrocatalyst Activity and Selectivity for the Oxygen Reduction Reaction. *ChemElectroChem* **8 (13)**, 2467-2478 (2021).
3. Zamora Zeledón, J. A. *et al.* Tuning the electronic structure of Ag-Pd alloys to enhance performance for alkaline oxygen reduction. *Nat. Commun.* **12 (620) (1)**, 1-9 (2021).
4. Łukaszewski, M., Soszko, M. & Czerwiński, A. Electrochemical Methods of Real Surface Area Determination of Noble Metal Electrodes-an Overview. *Int. J. Electrochem. Sci* **11**, 4442-4469 (2016).
5. Rudi, S., Cui, C., Gan, L. & Strasser, P. Comparative Study of the Electrocatalytically Active Surface Areas (ECSAs) of Pt Alloy Nanoparticles Evaluated by Hupd and CO-stripping voltammetry. *Electrocatalysis* **5 (4)**, 408-418 (2014).
6. Yoon, Y., Yan, B. & Surendranath, Y. Suppressing Ion Transfer Enables Versatile Measurements of Electrochemical Surface Area for Intrinsic Activity Comparisons. *J. Am. Chem. Soc.* **140 (7)**, 2397-2400 (2018).
7. Ong, S. P. *et al.* Python Materials Genomics (pymatgen): A robust, open-source python library for materials analysis. *Comput. Mater. Sci.* **68**, 314-319 (2013).
8. Persson, K. A., Waldwick, B., Lazic, P. & Ceder, G. Prediction of solid-aqueous equilibria: Scheme to combine first-principles calculations of solids with experimental aqueous states. *Phys. Rev. B* **85 (23)** (2012).
9. Atkins, P., de Paula, J. & Keeler, J. *Atkins' Physical Chemistry*. 9 edn, (Oxford University Press, 2010).
10. Haynes, W. M., Lide, D. R. & Bruno, T. J. *CRC Handbook of Chemistry and Physics: A Ready-Reference Book of Chemical and Physical Data*. 97 edn, (CRC Press, 2016).
11. Jung, M.-C., Kim, H.-D., Han, M., Jo, W. & Kim, D. C. X-Ray Photoelectron Spectroscopy Study of Pt-Oxide Thin Films Deposited by Reactive Sputtering Using O2/Ar Gas Mixtures. *Jpn. J. Appl. Phys.* **38 (Part 1, No. 8)**, 4872-4875 (1999).
